# Improvement of Predictive Ability by Uniform Coverage of the Target Genetic Space

**DOI:** 10.1534/g3.116.035410

**Published:** 2016-09-22

**Authors:** Daniela Bustos-Korts, Marcos Malosetti, Scott Chapman, Ben Biddulph, Fred van Eeuwijk

**Affiliations:** * C.T. de Wit Graduate School for Production Ecology and Resource Conservation (PE&RC), Wageningen, The Netherlands; † Biometris, Wageningen University and Research, The Netherlands; ‡ Commonwealth Scientific and Industrial Research Organisation (CSIRO) Agriculture, Queensland Bioscience Precinct, St. Lucia, Queensland 4067, Australia; § Department of Agriculture and Food, Western Australia, South Perth, Western Australia 6151, Australia

**Keywords:** genomic prediction, population structure, genetic space, training set, RKHS model, GenPred, Shared Data Resources, Genomic Selection

## Abstract

Genome-enabled prediction provides breeders with the means to increase the number of genotypes that can be evaluated for selection. One of the major challenges in genome-enabled prediction is how to construct a training set of genotypes from a calibration set that represents the target population of genotypes, where the calibration set is composed of a training and validation set. A random sampling protocol of genotypes from the calibration set will lead to low quality coverage of the total genetic space by the training set when the calibration set contains population structure. As a consequence, predictive ability will be affected negatively, because some parts of the genotypic diversity in the target population will be under-represented in the training set, whereas other parts will be over-represented. Therefore, we propose a training set construction method that uniformly samples the genetic space spanned by the target population of genotypes, thereby increasing predictive ability. To evaluate our method, we constructed training sets alongside with the identification of corresponding genomic prediction models for four genotype panels that differed in the amount of population structure they contained (maize Flint, maize Dent, wheat, and rice). Training sets were constructed using uniform sampling, stratified-uniform sampling, stratified sampling and random sampling. We compared these methods with a method that maximizes the generalized coefficient of determination (CD). Several training set sizes were considered. We investigated four genomic prediction models: multi-locus QTL models, GBLUP models, combinations of QTL and GBLUPs, and Reproducing Kernel Hilbert Space (RKHS) models. For the maize and wheat panels, construction of the training set under uniform sampling led to a larger predictive ability than under stratified and random sampling. The results of our methods were similar to those of the CD method. For the rice panel, all training set construction methods led to similar predictive ability, a reflection of the very strong population structure in this panel.

The key factor to progress in plant breeding is the number of genotypes that can be evaluated phenotypically (Cooper *et al.* 2014b). Unfortunately, field testing is slow and costly, forcing breeders to limit the number of genotypes that is phenotyped. Genomic prediction offers the potential to alleviate this limitation, allowing to broaden the pool of genotypes for selection, and thereby increasing selection intensity (Crossa *et al.* 2013; Windhausen *et al.* 2012) and efficiency of breeding programs (Heffner *et al.* 2010; Crossa *et al.* 2013; Windhausen *et al.* 2012; Hickey *et al.* 2014; Longin *et al.* 2015).

In genomic selection, genome-enabled genotypic or breeding values are calculated from genomic prediction models as sums of effects for large numbers of markers, often without explicitly testing individual marker–trait associations (Meuwissen *et al.* 2001). Genomic prediction models are developed for a target population of genotypes (TPG). The TPG describes the full collection of existing and future genotypes that is supposed to be suitably adapted to the environmental conditions defined by the target population of environments (Cooper *et al.* 2014a; Cooper and Hammer 1996; Comstock 1977).

Breeders have access to a sample from the TPG, the target sample. This sample of genotypes (or part of it) can be regarded as a calibration set for genomic prediction models when both phenotypic and marker data are available. To estimate the marker effects in prediction models, the calibration set is typically partitioned into a training set and a validation set. Marker effects are estimated on the training set of genotypes, and subsequently, genotypic values are calculated for all genotypes in the training and validation set. For accurate genomic prediction of the genotypic values in the validation set, training and validation sets should have similar genetic diversity, reflected in large kinship coefficients (Saatchi *et al.* 2011; Auinger *et al.* 2016). This condition is more likely to be met if the training set covers the whole genotypic, say genetic, space of the calibration set. As the calibration set is assumed to be a representative sample of the TPG, we also hope to cover the genetic space of the TPG. Therefore, a highly diverse TPG requires a larger training set size to capture the whole range of genetic diversity (Hayes *et al.* 2009).

Conventionally, genomic prediction literature uses random sampling as a strategy to split the calibration set into a training and a validation set (Burgueño *et al.* 2012; Crossa *et al.* 2010; Heslot *et al.* 2013; Schulz-Streeck *et al.* 2012; Riedelsheimer *et al.* 2012). In random sampling, genotypes belonging to the calibration set have equal probability to enter the training set. Hence, random sampling reproduces the genotypic frequencies of the calibration set, leading to a more dense coverage of those parts of the genetic space that are represented by a larger number of genotypes (Odong *et al.* 2013; Jansen and van Hintum 2007). Furthermore, we hypothesize that the heterogeneous coverage of the genetic space produced by random sampling leads to decreased predictive ability because part of the genetic diversity in the validation set is not well represented in the training set.

One strategy to improve the coverage of the genetic space is to use stratified sampling. In stratified sampling, the calibration set is divided into subpopulations and then a proportion of genotypes is randomly selected from each subpopulation (Guo *et al.* 2014; Albrecht *et al.* 2014; Janss *et al.* 2012; Daetwyler *et al.* 2012). However, subpopulations are sometimes not clearly defined or they are internally heterogeneous (Crossa *et al.* 2013). Thus, stratified sampling improves the coverage of the genetic space compared to random sampling, but it does not guarantee that all relevant genotypes are included in the training set.

The importance of an adequate representation of the genetic space for successful genomic prediction has been acknowledged in the recent literature. Rincent *et al.* (2012) assumed that predictive ability can be improved if genotypes in the training set are chosen in such a way that the precision of the contrasts between each genotype in the validation set and the mean of the calibration set is maximized. This can be achieved by maximizing the generalized coefficient of determination (CD). This method was further adapted by Isidro *et al.* (2015), who combined the method of Rincent *et al.* (2012) with stratified sampling. In this method (Isidro *et al.* 2015), the calibration set is first classified into subpopulations and then the CD mean criterion proposed by Rincent *et al.* (2012) is applied inside each subpopulation.

The methods proposed by Rincent *et al.* (2012) and by Isidro *et al.* (2015) rely on the variance components estimated from phenotypic data to choose genotypes for the training set. Although training set composition is not very sensitive to changes in variance components, some small differences in the genotypes allocated to the training set could be observed from trait to trait due to trait heritability differences (Rincent *et al.* 2012).

A statistically attractive strategy to increase the genetic similarity between training and validation sets is to uniformly cover the genetic space of the population of genotypes. Uniform coverage of the genetic space as a criterion for choosing members of the training set has the advantage of purely genotypic information being sufficient, without requiring phenotypic information (Jansen and van Hintum 2007; Odong *et al.* 2011). This principle is well known in the genetic resources literature, where it is used to define germplasm core collections (Odong *et al.* 2013). Here, we interpret the core collection as a training set because both of them, core collection and training set, are a subset of genotypes that aim at representing the genetic diversity present in a larger population.

Once the training set has been constructed, the next task is to identify a suitable prediction model. A large range of prediction models have been proposed, and they differ in two main aspects. The first aspect is the weight that models assign to specific genomic regions. If large QTL are present, predictive ability might benefit from modifying the common assumption that all marker effects come from a common normal distribution (Hayes *et al.* 2009). Hence, depending on the trait genetic architecture, it might be convenient to give more importance to genomic regions with large effects (Crossa *et al.* 2013; Daetwyler *et al.* 2010; Speed and Balding 2014; Hayes *et al.* 2009; Bernardo 2010).

The second aspect is whether the model accounts only for additive genetic effects, or also for nonadditive effects (Langer *et al.* 2014; Reif *et al.* 2011; Kippes *et al.* 2014; Stange *et al.* 2013). The GBLUP model proposed by Meuwissen *et al.* (2001) can be extended to separately account for nonadditive genetic effects (Oakey *et al.* 2006). However, the model proposed by Oakey *et al.* (2006) is computationally demanding. A less demanding model option for various types of nonadditive effects is the class of Reproducing Kernel Hilbert Space (RKHS) models, for example, with a Gaussian Kernel (Gianola and van Kaam 2008; Piepho 2009; Jiang and Reif 2015). The advantage of RKHS models is that they can be used across a spectrum of genetic architectures (de los Campos *et al.* 2009).

Given the importance of population structure and trait genetic architecture for effective implementation of a genomic prediction strategy, the objectives of this paper were (i) to compare strategies to define the training set, and (ii) to compare the predictive ability for models with explicit QTL with the predictive ability of GBLUP and RKHS models.

## Materials and Methods

### Data

To compare the strategies for training set construction and prediction models, we used four genotype panels that differed in the amount of population structure (Flint and Dent maize panels, and a wheat and rice panel).

### Maize

The maize data consisted of a Flint panel crossed with a Dent tester (F353) and of a Dent panel crossed with a Flint tester (UH007) to produce hybrid progeny for phenotypic evaluation, published by Rincent *et al.* (2014b). Both panels were composed of lines aiming at best representing the diversity of Flint and Dent maize in Northern Europe. The panels included commercially used inbred lines created from open pollinated varieties, and lines recently developed by public institutes or, in the case of the Dent panel, private companies.

The Dent panel consisted of 276 genotypes, whereas the Flint panel had 259 genotypes. Both panels were evaluated in field trials in Germany, France and Spain during 2010 and 2011. In this paper, we used the adjusted means of tasseling date, silking date and dry matter yield for each genotype across all environments [Supplemental Materials 12 and 13 in Rincent *et al.* (2014b)]. Tasseling and silking date were expressed as growing degree days after sowing, considering a base temperature of 6°, using the mean daily air temperature measured in each environment.

Both panels were characterized genotypically with the Illumina maize SNP50 BeadChip described in Ganal *et al.* (2011). From this set, we used only the markers that were developed by comparing the sequences of nested association mapping founder lines [PANZEA SNPs, Gore *et al.* (2009); Rincent *et al.* (2012)]. Individuals which had marker missing rate and/or heterozygosity higher than 0.10 and 0.05, respectively, were eliminated. Missing marker genotypes (below 2% in both panels) were imputed with the software BEAGLE. Markers with minor allele frequency lower than 0.05 were eliminated, leading to 28,304 PANZEA markers for the Dent panel, and 25,578 PANZEA markers for the Flint panel (Rincent *et al.* 2014b).

### Wheat

This wheat panel was constructed to represent flowering time variation present in Australian wheat germplasm. Phenotypic data corresponded to the adjusted means across environments for yield and heading date of 149 genotypes observed during 2009. Yield was observed at eight locations, whereas heading date was observed at six locations in the Australian wheat belt. Genotypes were characterized with 4295 SNPs, from which four SNPs were at the position of major genes regulating phenology (Ppd-D1, Vrn-A1, Vrn-B1, Vrn-D1). Missing markers were replaced by imputed genotypic data using the missForest package in *R*, following the methodology explained in Bogard *et al.* (2014). One marker was discarded as it showed >25% missing data, 39 markers were removed as they were monomorphic on this panel, and 431 were discarded because they had a minor allele frequency lower than 0.05. This led to 3754 markers for further analysis. Wheat genotypic and phenotypic data are available in Supplemental Material, File S1, File S2, and File S3.

### Rice

The rice data consisted of 413 diverse accessions of inbred lines from 82 countries. This data set is publicly available at http://www.ricediversity.org. Phenotypes consisted of plant height, seed number per panicle and flowering time in Arkansas. Genotypes that were too similar to each other (causing the relationship matrix to be singular) or that had a missing phenotype, were removed, leaving 350 genotypes for the analysis. The panel was genotyped with a 44-K SNP chip. After filtering, 36,091 markers were retained in the published data set. From this set of markers we discarded those that had >5% of missing values. The remaining missing marker scores were imputed with the software BEAGLE. Markers with minor allele frequency lower than 0.05 (considering only the phenotyped lines) were eliminated, leading to 26,259 markers.

### Characterization of the population structure

Population diversity was explored by principal component analysis of the identity by state (IBS) matrix among genotypes, calculated from molecular markers (Equation 1). This IBS calculation method indicates the proportion of shared alleles between genotypes.

$$A^{IBS}=\frac{GG^{\prime }+G_{2}G_{2}^{\prime }}{K}$$ (1)

In Equation 1, *G* is a genotype by marker matrix of marker scores, with 0 and 1 as scores for the homozygotes and 0.5 for the heterozygotes. *K* is the total number of markers and $G_{2}=1-G,$ where 1 is a matrix of ones.

The number of subpopulations present in each data set was determined with the Tracy–Widom statistic, following Patterson *et al.* (2006). Here, the number of subpopulations equals the number of significant principal components, plus one. Genotypes were qualitatively assigned to the subpopulation using the STRUCTURE software (Pritchard *et al.* 2000) and with the number of groups as determined by the Tracy–Widom statistic. To get an impression about population differentiation, the Fst statistic was calculated following Weir (1996) using a self-coded program in GenStat v.17 (VSN-International 2015).

### Training and validation sets

To split the calibration sets into a training and a validation set, we used the following five methods:

**Uniform coverage of the genetic space (U)** In U, we used the methodology proposed by Jansen and van Hintum (2007). This method consists of the following steps, which are applied to the list of all genotypes contained in the panel ($P_{1}$): (1) Molecular markers are used to calculate identity by state among all genotypes in $P_{1}$ (IBS, Equation 1). (2) The first entry of the training set ($T_{1}$) is sampled at random from the panel. Genotypes with a distance to $T_{1},$ smaller than a sampling radius *r*, are discarded from the training set. The new list of candidate genotypes is called $P_{2}.$ The genotypes that are discarded are stored in a list called $D_{1}.$ (3) The second entry of the training set is sampled at random from $P_{2}$ and it is called $T_{2}.$ Genotypes with a distance to $T_{2}$ smaller than the sampling radius *r* are discarded from the list of genotypes. This process is repeated until all the genotypes have been included in the training set $T_{n},$ or in the list of discarded genotypes ($D_{n}$). U is implemented in the “sampling” method of the GenStat procedure QGSELECT (VSN-International 2015).

**Figure 1:**
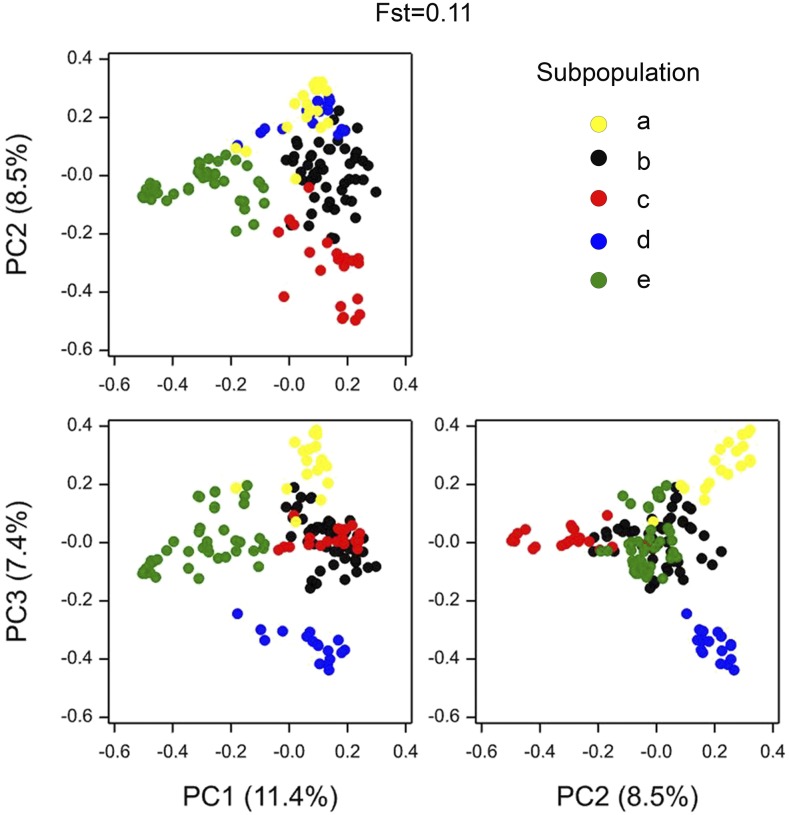
Scatter plots for principal components representing IBS matrix of the Flint panel. Symbol color represents each of the five subpopulations.

The sampling radius used in step (2) was obtained empirically. The size of this radius depends on the training set size one aims at. If the desired training set size is larger, the sampling radius becomes smaller. The target *r* is obtained by slowly decreasing its values until the number of sampled genotypes is greater than or equal to the target sample size, following Figure 1 in Jansen and van Hintum (2007).

**Stratified sampling with uniform coverage of the genetic space (SU)** In SU, prior information about the grouping of the genotypes was supplied. In this method, an extra restriction was added to the distance restriction. Genotypes are discarded when they are within the sampling radius and they belong to the same group (*i.e.*, they are included in the training set when they are within the sampling radius, but they belong to a different group). This method ensures that each group is represented by at least one genotype.
**Generalized coefficient of determination (CD)** The generalized coefficient of determination was used as a criterion to select genotypes for the training set in such a way that the precision of the prediction of the difference between the value of each individual in the validation set and the mean of the total calibration set is maximized (Rincent *et al.* 2012). Briefly, the precision is maximized when the generalized coefficient of determination (CD, Equation 2) is maximized.
  $$CD\left(c\right)=diag\left[\frac{c^{\prime }\left(A^{AB}-\lambda {\left(Z^{\prime }MZ+\lambda {\left(A^{AB}\right)}^{-1}\right)}^{-1}\right)c}{c^{\prime }A^{AB}c}\right]$$ (2)

In Equation 2, *c* is a matrix of the contrasts between each individual in the validation set and the mean of the calibration set, *M*, is an orthogonal projector of the subspace spanned by the columns of the design matrix of the fixed effects, *X*, (in our case, only the intercept): $M=I-X{\left(X^{\prime }\;X\right)}^{-}X^{\prime }.$ *λ* is the ratio between the residual and the additive genetic variance. For Flint and Dent, we calculated *λ* from the heritability estimates reported by Rincent *et al.* (2014b). For wheat heading time and yield, we used an estimate for *λ* calculated from the phenotypic data ($h^{2}$= 0.95 for heading time and $h^{2}$= 0.89 for yield). No heritability estimate was available for rice. Thus, we arbitrarily used 0.85 for the three rice traits.

$A^{AB}$ is the realized additive genetic relationship matrix calculated from all molecular markers along the whole genome following the equation proposed by Astle and Balding (2009), with as typical entry for the relationship between genotypes *i* and *j*:

$$A_{ij}^{AB}=\frac{1}{K}\sum_{k=1}^{K}\frac{\left(G_{ik}-2p_{k}\right)\left(G_{jk}-2p_{k}\right)}{2p_{k}\left(1-p_{k}\right)}$$ (3)

where $G_{ik}$ is a marker score that can take the value 2, 1, or 0 for genotype *i* at marker *k*, and $p_{k}$ is the allele frequency of marker *k*. The matrix above was calculated using the “realizedAB” option in the “kin” function of the Synbreed package (Wimmer *et al.* 2012).

The optimization algorithm used by Rincent *et al.* (2012) to construct the training set was implemented in R3.2.1. Briefly, at each step, one genotype in the training set is exchanged by one genotype in the validation set. This exchange is accepted if CD is increased and is rejected otherwise. The algorithm was allowed to iterate until the CD did not change anymore (800 times was enough to reach stability in all data sets).

**Stratified random sampling (S)** In S, the number of sampled genotypes depended on the logarithm of the subpopulation size, following Franco *et al.* (2005) and Malosetti and Abadie (2001).
  $$n_{t,s}=n_{t}\frac{\log\left(n_{s}\right)}{\sum_{s=1}^{S}\log\left(n_{s}\right)}$$ (4)

In Equation 4, $n_{t,s}$ is the number of genotypes to be sampled from subpopulation *s* into the training set, *S* is the number of subpopulations, $n_{t}$ is the total size of the training set we want to construct, and $n_{s}$ is the number of individuals belonging to subpopulation *s* in the calibration set. Within the subpopulations, genotypes were sampled at random.

**Random sampling (R)** In strategy R, the training set was sampled at random, so each genotype in the calibration set had equal probability of being included in the training set.

One hundred independent realizations of each of the five sampling strategies U, SU, CD, S, and R were generated for each calibration set. Each of the training sets (sampled genotypes) was used for QTL detection and as a training set for the prediction models.

### Characterization of the training sets

To characterize the connection between the training and the validation set, we calculated the distance between each genotype in the validation set and the nearest entry in the training set, following the method *Average distance between each accession and the nearest entry (A*–*NE)* in Odong *et al.* (2013). Here, we interpret the core collection in that paper, consisting of entries, as a training set. Core collection entries and training set members form a subset of genotypes that aim at representing a larger collection of genotypes. The set of accessions from which a core collection is created, we interpret to represent a calibration set. The distance between a genotype in the validation set and the nearest genotype in the training set [or core collection in Odong *et al.* (2013)] was calculated as (1−IBS). The empirical distribution of these distances was plotted for each training set construction method.

To obtain an impression of how each subpopulation is represented in the training set, we calculated the proportion of genotypes from each subpopulation that is included in the training set. The mean IBS in each subpopulation was used to relate the sampling proportion to the genetic diversity in each subpopulation.

### QTL detection

Training sets obtained by U, SU, CD, S or R sampling of the genotype panel were used to identify QTL that became part of the prediction model. QTL were identified by a genome-wide association mapping scan (GWAS), following Equation 5.

$$y_{i}=\mu +x_{ik}\alpha_{k}+G_{i}+e_{i}$$ (5)

In Equation 5, $y_{i}$ stands for the phenotype of genotype *i*, *μ* is the intercept, $x_{ik}$ is a vector that represents information of genotype *i* at marker *k* (0 and 2 for homozygous and 1 for heterozygous), and $\alpha_{k}$ is the additive QTL effect (fixed) for marker *k*. $G_{i}$ represents a polygenic effect for genotype *i*, and $e_{i}$ is the nongenetic residual $\left(e_{i}∼N\left(0,\sigma_{e}^{2}\right)\right)$. The distribution of $G_{i}$ is $∼N\left(0,A\sigma_{g}^{2}\right).$ *A* is the additive genetic relationship matrix calculated from the molecular marker information as in Rincent *et al.* (2014a). In this method, a specific *A* is calculated for each linkage group by excluding the markers on that particular linkage group. A significance threshold equivalent to a genome-wide significance level of 0.01 was used for the four data sets, following the Li and Ji (2005) multiple-testing correction. We performed the GWAS as implemented in GenStat 17th edition (VSN-International 2015).

### Prediction models

The following prediction models were used:

#### QTL:

$$y_{i}=\mu +\sum_{q\in Q}\left(x_{iq}\alpha_{q}\right)+e_{i}$$ (6)

In Equation 6, *μ* is the intercept, $\sum_{q\in Q}\left(x_{iq}\alpha_{q}\right)$ is for genotype *i* the sum of (random) QTL effects belonging to the QTL set *Q*, where these QTL were identified in a preliminary GWAS scan. Effects for each QTL were allowed to have their own distribution ($\alpha_{q}∼N\left(0,\sigma_{q}^{2}\right)$), and $e_{i}$ is the residual ($e_{i}∼N\left(0,\sigma_{e}^{2}\right)$).

#### GBLUP:

$$y_{i}=\mu +G_{i}+e_{i}$$ (7)

In Equation 7, *μ* is the intercept and $G_{i}$ represents the random genotype effects that follow a distribution $G_{i}∼N\left(0,A^{AB}\sigma_{g}^{2}\right).$ $A^{AB}$ is the additive relationship matrix, following Astle and Balding (2009) (Equation 3). The predictions were calculated using GenStat 17th edition (VSN-International 2015).

#### QGBLUP:

$$y_{i}=\mu +\sum_{q\in Q}\left(x_{iq}\alpha_{q}\right)+G_{i}+e_{i}$$ (8)

The model in Equation 8 combines the QTL and GBLUP model. Again, *μ* is the intercept, $\sum_{q\in Q}\left(x_{iq}\alpha_{q}\right)$ is the sum of random QTL effects from the QTL set *Q* for genotype *i*, with each of the QTL effects having proper variance component, $\alpha_{q}∼N\left(0,\sigma_{q}^{2}\right).$ The polygenic effects $G_{i}$ are assumed to follow a distribution $G_{i}∼N\left(0,A^{ABm}\sigma_{g}^{2}\right).$ $e_{i}$ is the residual $\left(e_{i}∼N\left(0,\sigma_{e}^{2}\right)\right)$. $A^{ABm}$ corresponds to a modified additive relationship matrix, calculated from all markers except those that were within a window of ±20cM around QTL. This precaution was taken to avoid accounting for the QTL effects both in the random QTL terms, and in the residual polygenic term. Again, predictions were calculated in GenStat 17th edition (VSN-International 2015).

#### RKHS:

The RKHS model is as the GBLUP model in Equation 7, but $G_{i}∼N\left(0,A^{*}\right).$ $A^{*}=\exp^{\left(-D/\theta \right)}$ represents the genetic relationship matrix, where *D* is a matrix with Euclidean dissimilarities among genotypes calculated from marker scores in the Synbreed package (Wimmer *et al.* 2012), and *θ* is a tuning parameter which determines how the covariance among individuals decays as a function of the genetic distance (Gianola and van Kaam 2008; Piepho 2009). An estimate for *θ* was obtained by fitting mixed models along a grid of values between 0.05 and 5. The *θ* value that provided the best predictive ability over a number of validation sets was used as the final *θ* value (de los Campos *et al.* 2010). The final *θ* value also showed the lowest AIC across the grid. The RKHS predictions were fitted by the REML procedure in GenStat v.17 (VSN-International 2015).

### Training set size

For maize, training sets contained 50, 70, 100, 150, or 200 genotypes [to match sample sizes chosen by Rincent *et al.* (2012)]. The wheat data had a limited panel size, so training set sizes of 50, 75, and 100 genotypes were used. Rice training sets had a size of 50, 100, 150, 200, or 300 genotypes to match the sizes used by Isidro *et al.* (2015).

### Predictive ability

Predictive ability was calculated as the Pearson correlation coefficient between observed and predicted phenotypes (Meuwissen *et al.* 2001). To evaluate whether predictive ability was driven by population structure, the Pearson correlation was calculated both across subpopulations, so ignoring population structure, and within the subpopulations, where it should be remarked that for smaller subpopulations no reliable estimates of predictive ability may be possible.

We wanted to study the influence of training set construction method, prediction model, and training set size on predictive ability. For each combination of these three factors, we calculated mean predictive ability across 100 training set realizations. We also calculated a standard error (SE). To comply with the normality assumption, correlations were analyzed on a transformed scale using Fischer’s *z* transformation, $z=\frac{1}{2}\left(\ln\left(\frac{1+r}{1-r}\right)\right)$, and means were back transformed using $r=\frac{\exp\left(2z\right)+1}{\exp\left(2z\right)-1}$ before reporting them.

### Data availability

The authors state that all data necessary for confirming the conclusions presented in the article are represented fully within the article.

## Results

We first explored population structure for the Flint, Dent, wheat, and rice panels. Subsequently, we investigated the properties of training sets constructed following the training set construction methods U, SU, CD, S, and R. Finally, we present the results of predictive abilities as defined by training set construction method and varying training set size and genomic prediction model. Predictive ability as estimated when ignoring population structure, *i.e.*, across subpopulations, was compared to predictive ability for individual subpopulations to establish the degree by which predictive ability was driven by population structure.

### Population structure

We present the panels ordered from the least to the most structured. Flint with an Fst statistic of 0.11 was the least structured panel; 5.96% of the total variation was explained by PC1 and 3.84% by PC2 (Figure 1). PC1 separated the Northern Flint genotypes from the other Flint genotypes, coinciding with what was reported by Rincent *et al.* (2012). The Tracy-Widom statistic indicated that four PCs were significant, suggesting five genetic groups. Although the separation between some groups is not visible in the three dimensions shown in Figure 1, groups were separated in higher dimensions.

The Dent panel with an Fst of 0.19 was slightly more structured than the Flint panel. A larger percentage of variation was explained by the first PCs (5.64% for PC1 and 4.62% for PC2, Figure S1). Five PCs were significant, thus, genotypes were classified into six subpopulations. The first PC separated the IODent from the non-IODent genotypes, the second PC separated the stiff-stalk from the non-stiff-stalk genotypes, and the third PC separated the D06 family from the rest. The remaining subpopulations were separated by PC4 and PC5.

For the wheat panel, Fst was 0.28 and four PCs were significant, indicating the presence of five subpopulations. PC1 (11.41%) tended to separate genotypes by their vernalization requirements, and PC2 (8.48%) tended to separate genotypes by their sensitivity to photoperiod (Figure S2).

**Figure 2:**
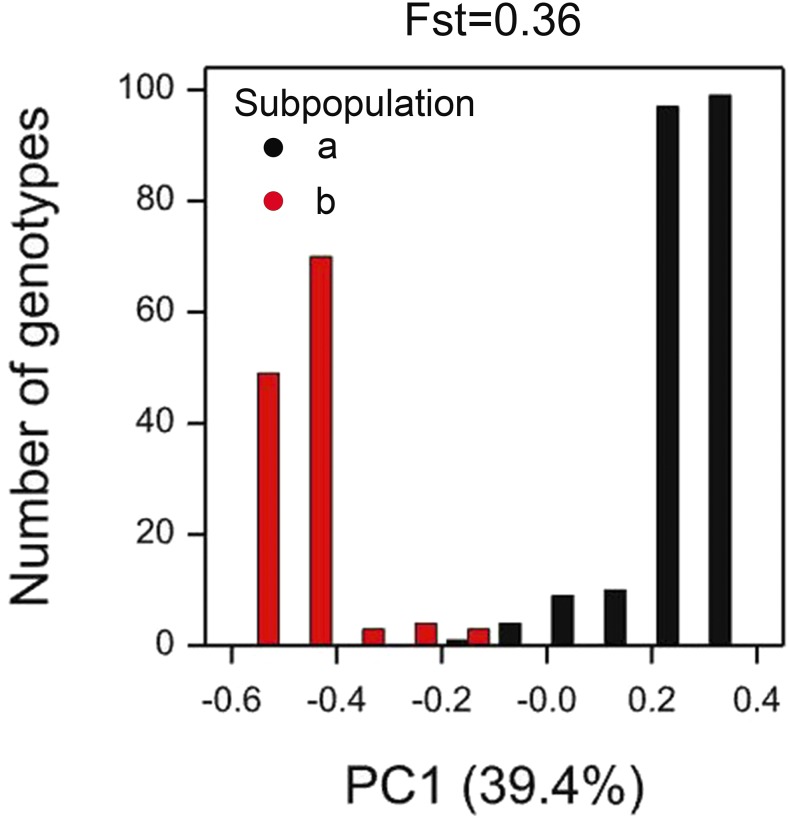
Histogram for the scores of the principal component representing the IBS matrix of the rice panel. Symbol color represents each of the subpopulations.

Rice was the most structured panel that we analyzed with an Fst of 0.36. Only the first PC was significant (39.41% of the variation), indicating two clearly distinguishable subpopulations (see Figure 2).

### Training and validation sets

In this section, we compare five methods to construct training sets from calibration sets (U, SU, CD, S, and R, see Table 1 for a description of the method abbreviations). Each individual calibration set is split into a training set and a validation set. For each combination of training set construction method, training set size, and genomic prediction model, 100 training sets were constructed, or drawn, from a calibration set.

**Table 1:** Abbreviations and descriptions for training set construction methods

Abbreviation	Description
U	Uniform coverage of the genetic space
SU	Stratified sampling with uniform coverage of the genetic space
CD	Generalized coefficient of determination(Rincent *et al.* 2012)
S	Stratified random sampling
R	Random sampling

#### Representation of subpopulations in training sets:

Random sampling of genotypes in the calibration set, *i.e.*, training set construction method R, to create a training set, will lead to a training set with proportional representation of subpopulations. In Table 2 we express the abundance of genotypes coming from a particular subpopulation when using training set construction methods U, SU, CD, and S relative to the abundance for that subpopulation as realized by application of training set construction method R. For all panels it held that large and diverse subpopulations were over-represented in the training sets created by application of U, SU, and CD in comparison to R. The lowest diversity subpopulations were always under-represented when using U, SU, and CD. Subpopulation affected representation in an expected way for the Dent panel and rice panel for the comparison of S *vs.* R, that is, larger subpopulations were under-represented and smaller subpopulations were over-represented. For the Flint and wheat panel the relationship between representation and subpopulation size was not clear. In conclusion, for U and SU, a relatively larger number of genotypes was allocated to the training set from those parts of the genetic space that were more diverse. CD behaved comparably to U and SU for all panels.

**Table 2:** Subpopulation size in the calibration set, genetic diversity (Div=1−median IBS) and number of calibration set genotypes assigned to the training set, expressed as a percentage of the number realized by random sampling

Flint, 200							Dent, 200							Wheat, 100							Rice, 300						
	Size	Div.	U	SU	CD	S		Size	Div.	U	SU	CD	S		Size	Div.	U	SU	CD	S		Size	Div.	U	SU	CD	S
a	50	0.26	−35	−25	−30	13	a	17	0.26	−61	−53	−42	38	a	17	0.18	−20	−13	−14	−9	a	220	0.17	−8	−8	−4	0
b	30	0.30	−5	12	−7	−18	b	45	0.28	−33	−31	−25	10	b	19	0.25	−5	−1	−4	−8	b	129	0.31	14	14	7	0
c	55	0.33	8	6	8	3	c	13	0.31	−1	−4	−10	35	c	41	0.31	−28	−24	2	2							
d	30	0.34	1	8	4	−1	d	38	0.32	−11	−13	−14	22	d	21	0.35	5	4	−3	6							
e	94	0.39	15	4	13	−3	e	40	0.36	−3	−4	−5	22	e	51	0.40	22	14	14	0							
							f	123	0.38	25	24	22	−27														

For the description of the training set construction methods U, SU, CD, S, and R, see Table 1.

#### Distance between validation set and training set:

Our objective was to evaluate methods for training set construction that provide a more homogeneous coverage of the genetic space and that reduce the genetic distance between genotypes in the validation set and those in the training set. The underlying rationale is that the lower the genetic distance (larger genetic relatedness) between validation and training sets, the better the predictive ability in the validation set is expected to be.

**Figure 3:**
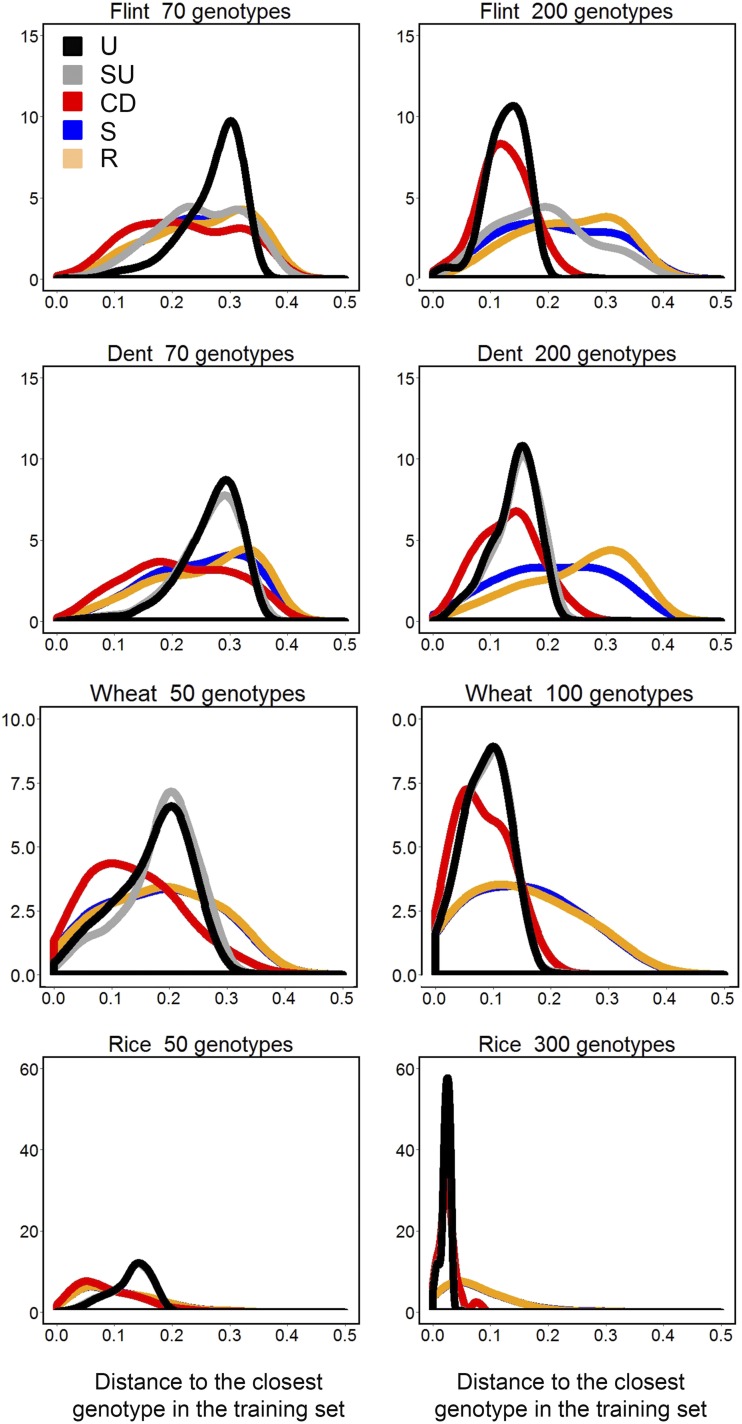
Distribution of genetic distances (distance=1−IBS) between validation set genotypes and the closest genotype in the training set (summed over 100 sampling events). For the description of the training set construction methods U, SU, CD, S, and R see Table 1.

Figure 3 shows the distribution of distances of validation set genotypes to the closest training set genotype, with distance=1−IBS, summed over all 100 realizations of the training set. A broad distribution indicates high heterogeneity of distance, *i.e.*, some validation genotypes are close to the training set, whereas others are distant. Our objective was to construct a training set that is on average close to the validation set with little variation between validation genotypes, reflected in a narrow distribution.

At all training set sizes, SU and U had a narrower distribution than CD, S, and R, showing that training set samples created by SU and U achieve a homogeneous coverage of the genetic space and that these sampling outcomes are consistent from realization to realization.

At small training set size, the median and the maximum genetic distance between genotypes in the validation set and those in the training set was similar for U, SU, S, and R. Only CD showed a smaller median distance, compared to the other four methods, especially for wheat and rice (Figure 3 and Table S1).

At larger training set sizes, the methods CD, U, and SU showed smaller distances between genotypes in the validation and training sets, compared to S and R (Figure 3 and Table S1). CD coincided with U and SU for the modal genetic distance, but tended to have a broader distance distribution. This broader genetic distance distribution implies that while on average CD, U, and SU are similar, CD tends to achieve a less homogeneous coverage of the genetic space, when compared to U and SU.

Incorporating *a priori* defined subpopulations into the genetic distance sampling, SU *vs.* U, had only a small effect for the least structured panels, Flint and Dent. For those panels, U showed a slightly narrower distribution than SU. This difference was most relevant at small sample sizes. In the case of more structured populations (wheat and rice), the incorporation of *a priori* subpopulation information into the sampling process did not change the distribution of genetic distances between validation and training sets. This means that as a desirable feature of our U method population substructure, whether subtle or not, it will automatically be accounted for in the construction of the training set.

#### QTL detection in the training set:

The number of detected QTL increased with training set size (Table S2, Table S3, and Table S4). At training set sizes smaller than 100 genotypes, the number of sets in which QTL were detected was very small and their positions changed across training sets. For training set sizes of 100 genotypes or larger, CD, U, and SU produced a larger number of QTL than S and R.

In the case of the Flint panel, most consistent QTL were detected on linkage group 1 for tasseling, silking and yield (Table S2). For Dent, QTL were detected most often on linkage groups three and eight for tasseling and silking and in linkage group 5 for yield (Table S3).

Very few QTL for grain yield were detected in the wheat panel. For heading time, large QTL for photoperiod and vernalization requirements appeared only at larger sample sizes, reflecting that the population was too small for QTL detection in the training set. However, given that the population was characterized for loci that are known to be relevant for vernalization and photoperiod sensitivity, we decided to include these four loci in all the QGBLUP and QTL models for heading date.

For the rice panel, the most consistent QTL for plant height was detected on linkage group 1 (Table S4). When using the methods U, SU, and CD, an important proportion of the training sets showed a QTL on linkage groups 2 and 6 at larger training set sizes. For seed number, a consistent QTL was detected for a training set size of 300 genotypes on linkage group 12. For flowering date, the most consistent QTL were detected on linkage groups 3 and 5. Again, these QTL were more often detected with U, SU, and CD, than with S and R.

#### Predictive ability in the validation set, ignoring subpopulations:

First, we present predictive ability as calculated on all genotypes in the validation set, pooling validation genotypes across subpopulations. To investigate the influence of the subpopulations on the accuracy, we have also calculated within subpopulation prediction abilities (see below).

**Figure 4:**
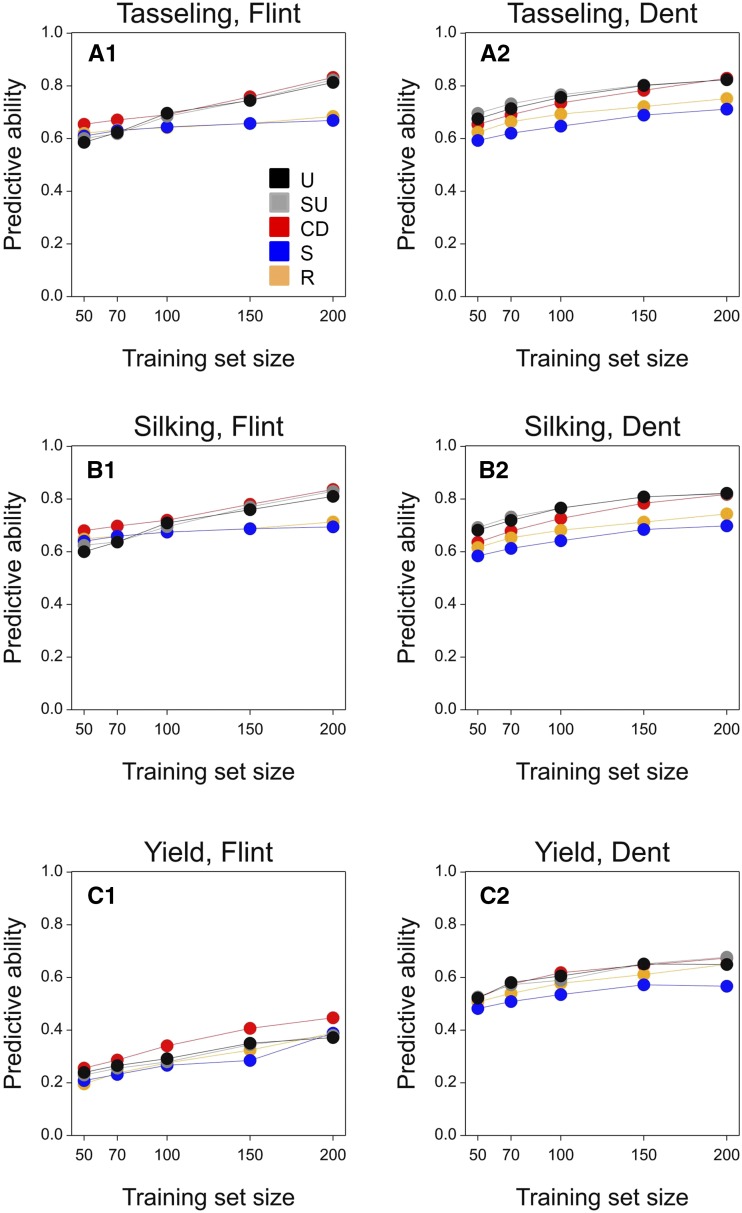
Predictive ability for the Flint and Dent panels as a function of training set size, using the RKHS model. The mean standard error for predictive ability was 0.001. For the description of the training set construction methods U, SU, CD, S, and R see Table 1.

**Figure 5:**
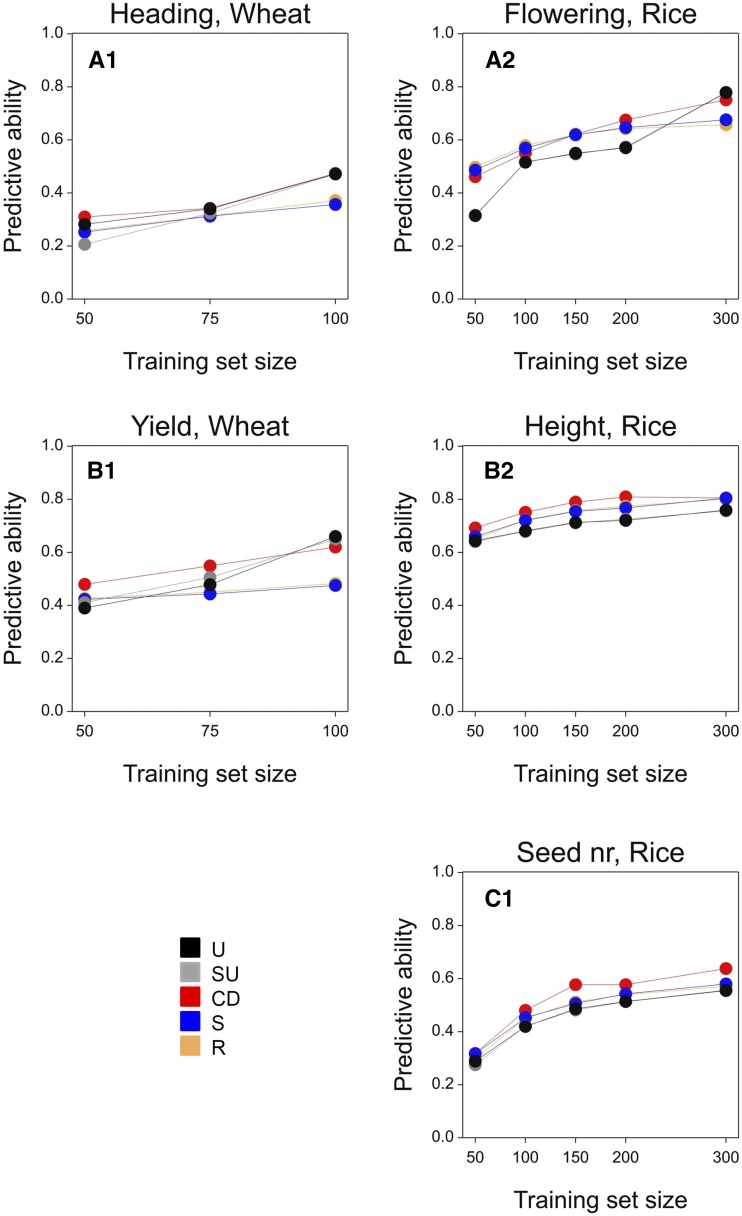
Predictive ability for the wheat and rice panels as a function of training set size, using the RKHS model. The mean standard error for predictive ability was 0.001. For the description of the training set construction methods U, SU, CD, S, and R see Table 1.

In the Flint, Dent, wheat, and rice panel, as expected, the relative predictive ability of methods depended on the training set size (Figure 4 and Figure 5). While at small training set sizes, differences between all methods were minor, at larger training set sizes, methods that reduced the distances between the validation and the training set (*i.e.*, U, SU, and CD) showed a clear improvement compared to S and R with an absolute increase in predictive ability of between 0.10 and 0.25.

Prediction models differed in predictive ability (Table 3, Table 4, Table 5, and Table 6). For the Flint, Dent, and rice panels, RKHS, GBLUP, and QGBLUP showed a larger predictive ability than the QTL model. This indicates that the evaluated traits were regulated by a large number of loci (Table 3, Table 4, and Table 6). For the same reason, including QTL in a separate model term (QGBLUP) was not advantageous over GBLUP. The comparable results of RKHS and GBLUP indicate that nonadditive genetic effects were not so relevant for the analyzed traits in the Flint, Dent, or rice panels.

**Table 3:** Predictive ability for the Flint panel, using a training set size of 200 genotypes

Model	U	SU	CD	S	R	SE
Silking, Flint, 200 genotypes						
QTL	0.514	0.531	0.468	0.373	0.378	0.010
GBLUP	0.810	0.830	0.836	0.695	0.713	0.009
QGBLUP	0.806	0.822	0.829	0.680	0.698	0.010
RKHS	0.819	0.832	0.835	0.684	0.706	0.009
Tasseling, Flint, 200 genotypes						
QTL	0.231	0.399	0.328	0.286	0.263	0.017
GBLUP	0.813	0.824	0.832	0.669	0.684	0.009
QGBLUP	0.784	0.800	0.798	0.619	0.635	0.017
RKHS	0.819	0.828	0.834	0.665	0.682	0.009
Yield, Flint, 200 genotypes						
QTL	0.287	0.443	0.187	0.067	0.130	0.021
GBLUP	0.372	0.381	0.447	0.388	0.388	0.021
QGBLUP	0.334	0.383	0.373	0.224	0.284	0.021
RKHS	0.380	0.378	0.444	0.373	0.377	0.010

For the description of the training set construction methods U, SU, CD, S, and R see Table 1. SE indicates the mean standard error across methods.

**Table 4:** Predictive ability for Dent, using a training set size of 200 genotypes

Model	U	SU	CD	S	R	SE
Silking, Dent, 200 genotypes						
QTL	0.461	0.471	0.396	0.409	0.367	0.008
GBLUP	0.822	0.820	0.818	0.698	0.744	0.007
QGBLUP	0.842	0.829	0.822	0.696	0.729	0.008
RKHS	0.818	0.814	0.805	0.621	0.678	0.007
Tasseling, Dent, 200 genotypes						
QTL	0.580	0.597	0.530	0.438	0.452	0.009
GBLUP	0.823	0.823	0.829	0.712	0.752	0.009
QGBLUP	0.839	0.832	0.826	0.707	0.741	0.009
RKHS	0.823	0.821	0.817	0.628	0.687	0.009
Yield, Dent, 200 genotypes						
QTL	0.416	0.395	0.403	0.241	0.300	0.009
GBLUP	0.649	0.677	0.674	0.567	0.650	0.007
QGBLUP	0.649	0.677	0.678	0.524	0.617	0.009
RKHS	0.621	0.646	0.655	0.523	0.603	0.007

For the description of the training set construction methods U, SU, CD, S, and R see Table 1. SE indicates the mean standard error across methods.

**Table 5:** Predictive ability for wheat, using a training set size of 100 genotypes

Model	U	SU	CD	S	R	SE
Wheat, heading, 100 genotypes						
QTL	0.303	0.301	0.336	0.382	0.351	0.009
GBLUP	0.472	0.472	0.474	0.357	0.371	0.009
QGBLUP	0.512	0.519	0.562	0.517	0.478	0.009
RKHS	0.632	0.611	0.592	0.421	0.419	0.009
Wheat, yield, 100 genotypes						
GBLUP	0.660	0.650	0.620	0.475	0.482	0.009
RKHS	0.699	0.679	0.654	0.538	0.517	0.009

For the description of the training set construction methods U, SU, CD, S, and R see Table 1. SE indicates the mean standard error across methods.

**Table 6:** Predictive ability for rice, using a training set size of 300 genotypes

Model	U	SU	CD	S	R	SE
Flowering, rice, 300 genotypes						
QTL	0.309	0.320	0.303	0.271	0.267	0.013
GBLUP	0.778	0.779	0.751	0.676	0.657	0.013
QGBLUP	0.766	0.770	0.728	0.673	0.653	0.013
RKHS	0.815	0.816	0.787	0.699	0.677	0.013
Height, rice, 300 genotypes						
QTL	0.379	0.379	0.301	0.361	0.366	0.014
GBLUP	0.759	0.756	0.805	0.804	0.800	0.011
QGBLUP	0.740	0.738	0.801	0.806	0.801	0.011
RKHS	0.785	0.779	0.806	0.790	0.788	0.011
Seed number, rice, 300 genotypes						
QTL	0.231	0.223	0.275	0.191	0.191	0.019
GBLUP	0.556	0.554	0.638	0.580	0.571	0.013
QGBLUP	0.479	0.467	0.582	0.515	0.519	0.019
RKHS	0.603	0.599	0.671	0.589	0.579	0.013

For the description of the training set construction methods U, SU, CD, S, and R see Table 1. SE indicates the mean standard error across methods.

Model ranking was slightly different for heading date in the wheat panel from that in Flint, Dent, and rice. In the case of heading date, QGBLUP led to larger predictive ability, compared to GBLUP and QTL (Table 5). This indicates that, for heading time in wheat, it is convenient to account separately for loci with large effects. However, RKHS showed a larger predictive ability than QGBLUP, reflecting that nonadditive genetic effects contribute to phenotypic variation of heading date. In the case of grain yield, no large QTL were consistently detected and therefore, we only used RKHS and GBLUP to predict this trait in wheat. As for heading, RKHS showed a larger yield predictive ability than GBLUP.

#### Predictive ability in the validation set, calculated within subpopulations:

We present predictive ability as calculated within subpopulations for the Flint, Dent, and rice panel. The wheat data were not included in this analysis because the panel was too small, and predictive ability within subpopulations could not be calculated reliably.

**Figure 6:**
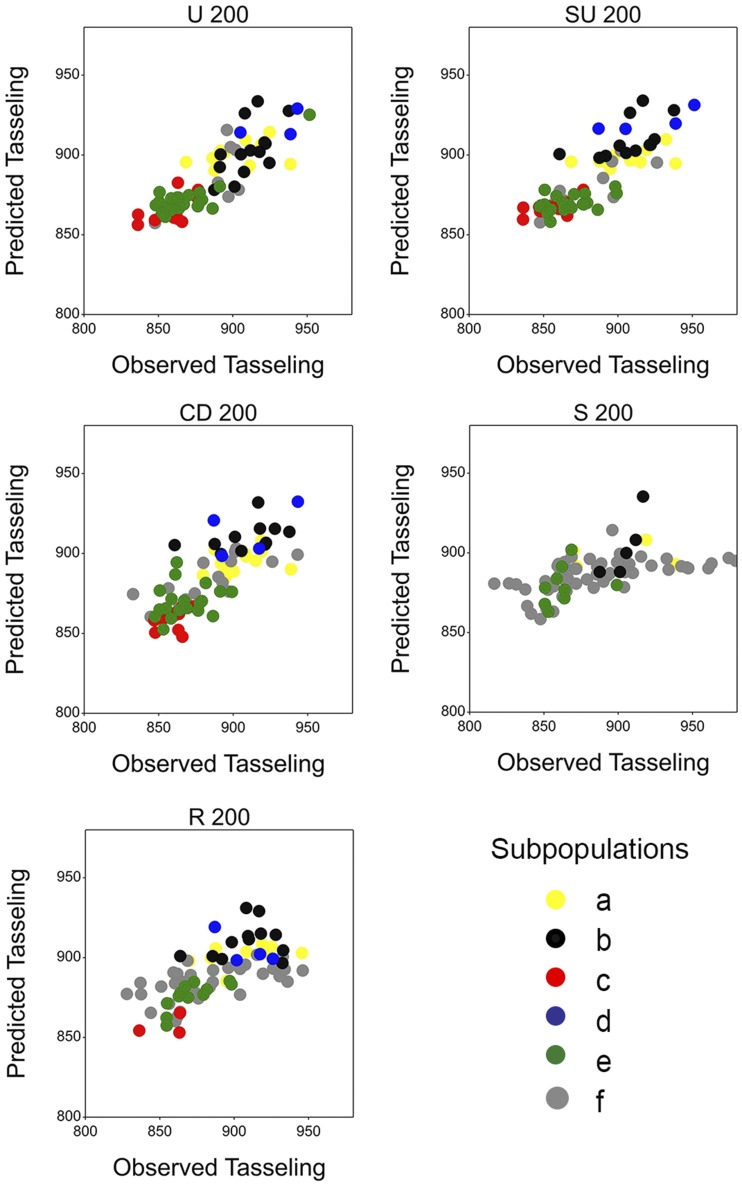
Relation between predicted and observed tasseling date for the Dent panel using the RKHS model and 200 genotypes. A single training set realization is shown for each training set construction method. Symbol color represents each of the six subpopulations.

Within subpopulations, training set construction methods generally maintained their ranking, compared to predictive ability calculated across subpopulations; U, SU, and CD were better than S and R (Table 7, Table S5, Table S6, Table S7, Table S8, and Table S9). This indicates that the improvement in predictive ability observed for U, SU, and CD was not driven by the subpopulations. This result can also be observed in the correlation plot between predicted and observed phenotypes. Figure 6 shows that the relation between predicted and observed trait values was similar within subpopulations and across subpopulations, demonstrating that predictive ability was not driven by population structure.

**Table 7:** Predictive ability within groups for Flint silking date, using a training set size of 200 genotypes

Flint, Silking date, 200 genotypes						
Subpop.	U	SU	CD	S	R	SE
QTL						
a	0.073	0.104	0.14	0.043	0.232	0.052
b	0.775	0.893	0.663	0.686	0.648	0.061
c	0.803	0.446	0.761	0.347	0.439	0.034
d	0.603	0.797	0.641	0.680	0.622	0.053
e	0.371	0.191	0.258	0.121	0.182	0.023
GBLUP						
a	0.485	0.588	0.485	0.395	0.577	0.031
b	0.625	0.867	0.656	0.579	0.638	0.039
c	0.850	0.331	0.908	0.449	0.575	0.028
d	0.802	0.726	0.860	0.501	0.534	0.043
e	0.666	0.563	0.727	0.634	0.563	0.019
QGBLUP						
a	0.452	0.597	0.509	0.420	0.552	0.038
b	0.611	0.867	0.625	0.654	0.647	0.048
c	0.864	0.402	0.899	0.489	0.561	0.034
d	0.705	0.736	0.803	0.631	0.574	0.053
e	0.737	0.603	0.714	0.523	0.512	0.023
RKHS						
A	0.578	0.576	0.519	0.266	0.554	0.031
B	0.625	0.959	0.627	0.554	0.629	0.039
C	0.807	0.354	0.877	0.523	0.609	0.028
D	0.760	0.753	0.859	0.509	0.582	0.043
E	0.732	0.559	0.732	0.633	0.554	0.019

For the description of the training set construction methods U, SU, CD, S, and R see Table 1. SE indicates the mean standard error across methods.

For the rice data, predictive ability within subpopulations was similar for all the training set construction methods, coinciding with the result observed for the predictive ability across subpopulations (Table S10, Table S11, and Table S12).

For all the panels, the ranking of prediction models with respect to within subpopulation predictive abilities coincided with that for across subpopulations; RKHS, GBLUP, and QGBLUP were similar (with minor differences in the ranking, depending on the panel), whereas the QTL model led to clearly lower predictive ability.

## Discussion

The main objective of this study was to assess the impact of five training set construction methods (U, SU, CD, S, and R) on predictive ability in the validation set. A secondary objective was to compare four prediction models that differ in the importance that they assign to specific genomic regions and in the type of genetic effects that they consider (additive/nonadditive). The training set construction methods and prediction models were evaluated at different training set sizes in four diversity panels. Predictive ability was calculated for the validation set in all the panels.

### Training set construction methods

Prediction of unobserved genotypes is possible provided that genotypes to be predicted are genetically similar to those that have been observed (Habier *et al.* 2010; Saatchi *et al.* 2011). Hence, a prerequisite to obtain large predictive ability is that the training set represents well the calibration set and that the calibration set represents well the TPG (Rincent *et al.* 2012; Crossa *et al.* 2013; Albrecht *et al.* 2014; Auinger *et al.* 2016).

Breeding populations are commonly structured. When population structure is present, genetic similarity is heterogeneous, because pairs of genotypes can belong to the same or different subpopulations. Random sampling from the calibration set reproduces its distributional properties without taking into account diversity differences across the genetic space (Jansen and van Hintum 2007). Thus, in structured populations, simple random sampling will not result in training sets that adequately represent the full genetic variation in the calibration set, leading to on average lower similarity between genotypes in the training and the validation set (Pszczola *et al.* 2012; Albrecht *et al.* 2011; Wientjes *et al.* 2016).

We showed that a more homogeneous coverage of the genetic space by applying the methods U and SU leads to smaller distances between genotypes in training and validation sets, and to a higher predictive ability. A uniformly covered genetic space also offers the potential to provide good predictive ability for new genotypes not belonging to the initial calibration set, provided that they are contained within the genetic space spanned by the initial calibration set.

Rincent *et al.* (2012) proposed to increase predictive ability by maximizing the precision of the contrast between each individual in the validation set and the mean of the calibration set (training and validation sets). This method was also successfully applied to genomic prediction in pea (Tayeh *et al.* 2015). Here, we show that CD, U, and SU are alternative methods that deliver comparable results because they all provide a training set that has a smaller genetic distance to the validation set. One of the advantages of U and SU is that no estimate of heritability is required. Thus, it resolves the unavoidable ambiguity when defining a training set for multiple traits with different heritabilities. A second advantage is that U and SU showed more consistency of training set sample properties revealed by a narrower distribution of distances between the validation and the training set, compared to CD, S, and R. The genotypes in the training set are at more constant distances, providing a more uniform coverage of the genetic space and larger predictive ability, even when the distribution of genotypic distances in the validation set is different from that in the training set. Furthermore, U and SU have the advantage that they are computationally easier and faster to apply than CD.

U, SU, and CD are methods that use genetic similarity/distance as a criterion to construct the training set. Thus, the set of markers used for distance calculation influences training set composition. One aspect that could be further explored is the convenience of considering only those genomic regions that influence the trait of interest, especially for traits regulated by a small number of loci. In the same vein, the presence of ascertainment bias in the marker set needs to be evaluated because it might modify the relative distances among genotypes, and, therefore, the training set composition. For that reason, we repeated all calculations for maize, using the full SNP50 BeadChip in place of the PANZEA marker set (results not shown). The relative distances among genotypes were highly comparable between those two marker sets (Frascaroli *et al.* 2012) and therefore we did not observe changes in the ranking of training set construction methods or prediction models for predictive ability.

### Prediction models

The main difference among prediction models is the relative importance assigned to specific loci as contrasted with the rest of the genome. It is therefore natural to expect that the degree of success of the different models depends on trait genetic architecture. This study dealt with yield, yield components (regulated by many loci with small effects), and with phenology traits. In the case of wheat, flowering time is regulated mainly by a few loci with large effect. However, despite the apparently simple genetic regulation of heading date favoring a QTL model, it is still beneficial to include a term that accounts for residual genetic variance. This result is in line with Zheng *et al.* (2013), who showed that flowering time in wheat is not only regulated by major genes for photoperiod and vernalization requirements, but also by a polygenic effect that influences earliness *per se*. In contrast, in the case of maize and rice, phenology and yield traits are regulated by many QTL (Buckler *et al.* 2009; Rincent *et al.* 2014b; Zhao *et al.* 2011). The more complex genetic architecture of maize and rice traits is in agreement with our findings of models using genome-wide information showing larger predictive ability than those using information from a few QTL (QTL prediction model).

The importance of considering trait genetic architecture when selecting the prediction model was also discussed by Daetwyler *et al.* (2010) and by Bernardo (2014), who simulated diverse traits that differed in the number of QTL explaining the genotypic variance. The authors observed that traits regulated by a small number of QTL tend to be predicted better by models that give a larger importance to QTL with large effects, compared to the GBLUP model. This result has also been observed for a set of human diseases regulated by few loci with different effect size, for which it was advantageous to include several random terms (Speed and Balding 2014). We are aware that the number of QTL included in our QGBLUP models contains an element of subjectivity because of the selection of a significance threshold to define when a locus enters the QTL list. Bernardo (2014) gave some guidelines about when to include the QTL in a separate model term.

Previous paragraphs discussed the convenience of separately accounting for additive loci, depending on their effect size. However, part of the genetic variance might be nonadditive. If the epistasis is simple (interaction between a few loci with large effects), it can be modeled as a QTL-interaction term (Malosetti *et al.* 2011). Unfortunately, in the case of the traits analyzed here, epistasis has been shown to be largely complex (Reif *et al.* 2011; Kippes *et al.* 2014). Langer *et al.* (2014) showed that epistasis for heading date in wheat can be dissected into at least 30 epistatic interactions, among which many of them did not correspond to interaction between large phenology genes. The results shown by Langer *et al.* (2014) coincide with the lack of improvement in predictive ability that we observed when we incorporated additional terms accounting for interaction among large phenology genes (results not shown). The RKHS model allows to account for epistatic interactions, without the need of specifying which genomic regions are responsible for this interaction (Crossa *et al.* 2010, 2013; Gianola and van Kaam 2008; Jiang and Reif 2015).

Traits and crops might also differ in the relative size of epistatic interactions (Langer *et al.* 2014; Reif *et al.* 2011; Spindel *et al.* 2015; Blanc *et al.* 2006). For example, a larger improvement was observed with the RKHS model for wheat data than for maize and rice. This result coincides with those of Endelman (2011) and Stange *et al.* (2013), who observed that the advantage of the RKHS model was large in the case of wheat grain yield, but it was small in the case of maize traits.

A further issue that needs to be considered in structured populations is the convenience of assuming constant or heterogeneous allele effects across subpopulations (Lehermeier *et al.* 2015; de los Campos *et al.* 2015). Models that allow for subpopulation-specific allele effects range from models that assume fully independent populations (effects estimated in each population separately), to more complex models that allow allele effects to be correlated across subpopulations (Lehermeier *et al.* 2015; Olson *et al.* 2012). In this paper, we focused on models that assume homogeneous effects. We also explored the idea of allowing for subpopulation-specific effects by fitting all the models to each subpopulation independently (not shown). However, models that allow for subpopulation-specific effects did not show a clear advantage over models with homogeneous effects, coinciding with Lehermeier *et al.* (2015), Schulz-Streeck *et al.* (2012), and Albrecht *et al.* (2011).

### Sample size

Sample size reduction inevitably leads to a larger probability of losing genotypes with extreme values for the trait of interest, thereby narrowing down the phenotypic trait range and the predictive ability. Our results showed a nonlinear decrease in predictive ability as a function of training set size. This nonlinear decrease of the predictive ability was also observed by Heffner *et al.* (2011), Zhao *et al.* (2012), and Rincent *et al.* (2012) and can be explained by the number of individuals, trait heritability, and the effective number of chromosome segments (Daetwyler *et al.* 2008, 2013).

When assessing the sampling methods in relation to sample size, U produced a more homogeneous representation of the genetic diversity of the original population, compared to S and R, leading to larger predictive ability. The fact that this advantage was maintained only at large sample sizes can be explained by the fact that, at smaller training set sizes, none of the training sets was able to provide enough information for an accurate estimation of genotypic effects.

### Conclusions

Training set construction methods that take into account the genetic diversity of the calibration set have higher predictive ability and are not sensitive to population structure in the calibration set: U, SU, and CD *vs.* S and R.
U and SU and CD produce comparable predictive abilities, but U and SU are simpler to calculate and require less computational cost and no phenotypic information in comparison to CD.
As expected, training sample size reduction led to lower predictive ability, but this reduction was stronger for the wheat and maize panels than for the rice panel.

## 

## Supplementary Material

Supplemental Material
