# Supplementary material for: Improvement of Predictive Ability by Uniform Coverage of the Target Genetic Space
Source: G3 (Bethesda). 2016 Sep 22;6(11):3733–47. doi: 10.1534/g3.116.035410 (PMC5100872; doi:10.1534/g3.116.035410)
Supplement: Supplemental Material [file supp_6_11_3733__index.html]

Improvement of Predictive Ability by Uniform Coverage of the Target Genetic Space — Supplemental Material 

# Improvement of Predictive Ability by Uniform Coverage of the Target Genetic Space

## Supplemental Material for Bustos-Korts *et al.*, 2016

**Files in this Data Supplement:**

- Table S1 - Median of the distance (1-IBS) between validation set genotypes and the closest genotype in the training set (over 100 sampling events). (.pdf, 14 KB)
- Table S10 - Rice Height predictive ability within groups using a training set size of 300 genotypes. (.pdf, 16 KB)
- Table S11 - Rice Flowering date predictive ability within groups using a training set size of 300 genotypes. (.pdf, 15 KB)
- Table S12 - Rice Seed number predictive ability within groups using a training set size of 300 genotypes. (.pdf, 71 KB)
- Table S2 - Flint number of QTLs with a genome-wide significant threshold p<0.01 (Li and Ji, 2005). (.pdf, 123 KB)
- Table S3 - Dent number of QTLs with a genome-wide significant threshold p<0.01 (Li and Ji, 2005). (.pdf, 139 KB)
- Table S4 - Rice number of QTLs with a genome-wide significant threshold p<0.01 (Li and Ji, 2005). (.pdf, 144 KB)
- Table S5 - Flint Tasseling date predictive ability within groups using a training set size of 200 genotypes. (.pdf 20 KB)
- Table S6 - Flint Yield predictive ability within groups using a training set size of 200 genotypes. (.pdf, 21 KB)
- Table S7 - Dent Silking date predictive ability within groups using a training set size of 150 genotypes. (.pdf, 21 KB)
- Table S8 - Dent Tasseling date predictive ability within groups using a training set size of 150 genotypes. (.pdf, 21 KB)
- Table S9 - Dent Yield predictive ability within groups using a training set size of 150 genotypes. (.pdf, 22 KB)
- Figure S1 - Representation of principal components analysis on the IBS matrix of the Dent panel. (.jpg, 951 KB)
- Figure S2 - Representation of principal components analysis on the IBS matrix of the wheat panel. (.jpg, 1 MB)
- File S1 - Wheat subpopulations and adjusted means for yield and heading date. (.csv, 4 KB)
- File S2 - Wheat genetic map. (.txt, 64 KB)
- File S3 - Wheat marker scores. (.txt, 2.22 MB)
