# Supplementary material for: Improvement of Predictive Ability by Uniform Coverage of the Target Genetic Space
Source: G3 (Bethesda). 2016 Sep 22;6(11):3733–47. doi: 10.1534/g3.116.035410 (PMC5100872; doi:10.1534/g3.116.035410)
Supplement: Supplemental Material [file supp_g3.116.035410_TableS1.pdf]

Table S1. Median of the distance (1-IBS) between validation set genotypes and the closest genotype in the training set (over 100 sampling events). For the description of the training set construction methods U, SU, CD, S and R see Table 1.

**Panel**	**Size**	**U**	**SU**	**CD**	**S**	**R**
Flint	70	0.29	0.24	0.22	0.26	0.25
	200	0.13	0.19	0.12	0.23	0.21
Dent	70	0.28	0.27	0.21	0.27	0.25
	200	0.15	0.15	0.14	0.27	0.21
Wheat	50	0.19	0.19	0.13	0.19	0.19
	100	0.09	0.09	0.07	0.14	0.15
Rice	50	0.13	0.13	0.08	0.09	0.09
	300	0.02	0.02	0.02	0.06	0.06
