# Supplementary material for: Improvement of Predictive Ability by Uniform Coverage of the Target Genetic Space
Source: G3 (Bethesda). 2016 Sep 22;6(11):3733–47. doi: 10.1534/g3.116.035410 (PMC5100872; doi:10.1534/g3.116.035410)
Supplement: Supplemental Material [file supp_g3.116.035410_TableS10.pdf]

Table S10. Rice Height predictive ability within groups using a training set size of 300 genotypes. For the description of the training set construction methods U, SU, CD, S and R see Table 1.

Plant height, rice, 300 genotypes						
**QTL**						
**Subpop.**	**U**	**SU**	**CD**	**S**	**R**	**s.e.**
a	0.216	0.219	0.181	0.160	0.139	0.038
b	-	0.099	-0.356	-	0.148	0.044
**GBLUP**						
**Subpop.**	**U**	**SU**	**CD**	**S**	**R**	**s.e.**
a	0.758	0.759	0.717	0.707	0.683	0.015
b	0.973	0.479	0.894	0.974	0.481	0.036
**QGBLUP**						
**Subpop.**	**U**	**SU**	**CD**	**S**	**R**	**s.e.**
a	0.741	0.744	0.683	0.691	0.670	0.015
b	0.971	0.501	0.889	0.970	0.518	0.036
**RKHS**						
**Subpop.**	**U**	**SU**	**CD**	**S**	**R**	**s.e.**
a	0.795	0.795	0.753	0.725	0.695	0.015
b	0.963	0.534	0.924	0.963	0.526	0.036
