# Supplementary material for: Improvement of Predictive Ability by Uniform Coverage of the Target Genetic Space
Source: G3 (Bethesda). 2016 Sep 22;6(11):3733–47. doi: 10.1534/g3.116.035410 (PMC5100872; doi:10.1534/g3.116.035410)
Supplement: Supplemental Material [file supp_g3.116.035410_TableS11.pdf]

Table S11. Rice Flowering date predictive ability within groups using a training set size of 300 genotypes. For the description of the training set construction methods U, SU, CD, S and R see Table 1.

**Flowering, rice, 300 genotypes**						
**QTL**						
**Subpop.**	**U**	**SU**	**CD**	**S**	**R**	**s.e.**
a	0.219	0.253	0.396	0.326	0.314	0.019
b	0.894	0.897	0.813	0.690	0.707	0.115
**GBLUP**						
**Subpop.**	**U**	**SU**	**CD**	**S**	**R**	**s.e.**
a	0.706	0.702	0.730	0.762	0.741	0.016
b	0.982	0.981	0.939	0.767	0.794	0.028
**QGBLUP**						
**Subpop.**	**U**	**SU**	**CD**	**S**	**R**	**s.e.**
a	0.685	0.680	0.720	0.752	0.728	0.016
b	0.981	0.980	0.944	0.789	0.812	0.028
**RKHS**						
**Subpop.**	**U**	**SU**	**CD**	**S**	**R**	**s.e.**
a	0.737	0.730	0.731	0.753	0.736	0.016
b	0.983	0.983	0.943	0.752	0.776	0.028
