# Supplementary material for: Improvement of Predictive Ability by Uniform Coverage of the Target Genetic Space
Source: G3 (Bethesda). 2016 Sep 22;6(11):3733–47. doi: 10.1534/g3.116.035410 (PMC5100872; doi:10.1534/g3.116.035410)
Supplement: Supplemental Material [file supp_g3.116.035410_TableS12.pdf]

Table S12 Rice Seed number predictive ability within groups using a training set size of 300 genotypes. For the description of the training set construction methods U, SU, CD, S and R see Table 1.

Seed number, rice, 300 genotypes					
		QTL			
**Subpop.**	**U**	**SU**	**CD**	**S**	**R**
**a**	0.246	0.228	0.310	0.244	0.258
**b**	-0.344	-0.523	0.025	0.041	0.076
		GBLUP			
**Subpop.**	**U**	**SU**	**CD**	**S**	**R**
**a**	0.508	0.505	0.527	0.563	0.555
**b**	0.956	0.963	0.834	0.656	0.630
		QGBLUP			
**Subpop.**	**U**	**SU**	**CD**	**S**	**R**
**a**	0.429	0.415	0.471	0.480	0.484
**b**	0.931	0.956	0.829	0.618	0.625
		RKHS			
**Subpop.**	**U**	**SU**	**CD**	**S**	**R**
**a**	0.562	0.557	0.584	0.582	0.569
**b**	0.968	0.972	0.841	0.665	0.645
