# Supplementary material for: Improvement of Predictive Ability by Uniform Coverage of the Target Genetic Space
Source: G3 (Bethesda). 2016 Sep 22;6(11):3733–47. doi: 10.1534/g3.116.035410 (PMC5100872; doi:10.1534/g3.116.035410)
Supplement: Supplemental Material [file supp_g3.116.035410_TableS2.pdf]

Table S2. Flint number of QTLs with a genome-wide significant threshold p<0.01 (Li and Ji, 2005). Columns 1-10 represent the linkage groups where the QTLs were located. Multiple QTLs can occur on a chromosome. For the description of the training set construction methods U, SU, CD, S and R see Table 1.

Tasseling

Size	Method	1	2	3	4	5	6	7	8	9	10
50	U			1		2				1	
	SU			1			3	1		4	
	CD	10			7			2	3	2	2
	S	9	4	9	10	3	3	4	7	2	2
	R	12	7	5	12	5	3	8	8	5	2
70	U		3			10			1		
	SU			4		4		1	1		
	CD	11	2	4		1	1	3	6	1	5
	S	19	1	1	6	1	2	2	3	9	1
	R	17	2	1	6	2	2	4	3	2	3
100	U		8			1		3	1	4	
	SU		1					3			
	CD	9		4	7				3		2
	S	23	3	2	4		2	3	1		3
	R	10	4	12	3	3		1	3	9	3
150	U	6		6				1		5	
	SU	33		2					1		
	CD	12		3	5				1		
	S	19	2	1	3			2	4	1	1
	R	22	2	2	3			1		2	
200	U	7		14							
	SU	28		12							
	CD	9		9							
	S	49	3	2						6	
	R	25	1	2	6			2		2	

Silking

Size	Method	1	2	3	4	5	6	7	8	9	10
50	U		1							1	
	SU					1		1		2	
	CD	16	4	1	9			3	3	2	7
	S	13	9	8	6	2	2	2	8	3	2
	R	13	6	7	13	5	2	4	7	4	2
70	U		8				1				
	SU		6	3		1		1	2	1	
	CD	5	2	2	5			1	6		8
	S	20	1	14	5	3	2	2	2	7	3
	R	15	4	2	7	1	3	2	5	1	2
100	U		17					7	1	9	
	SU		7	2				3		2	
	CD	11	1	5	5				2		
	S	28	4	2	3		1	2		1	1
	R	15	4	13	5	1		1	4	5	3
150	U	25	1	6				9		15	
	SU	84	2	2				5		2	
	CD	15		4	2				1	1	
	S	31	3		2		2		2	1	2
	R	17	4	3	2		1	1	2	1	
200	U	75		2						3	
	SU	100									
	CD	53		9				2		4	
	S	83	8	2	1				1	7	
	R	48	5	3	3		1			4	1

Yield

Size	Method	1	2	3	4	5	6	7	8	9	10
50	U	9									
	SU				2					7	
	CD	2	3			1			1		
	S	3			5	2					3
	R	2		1	4					1	1
70	U	2	1								
	SU	13								1	
	CD	1	4		1	3	1	1	2		1
	S	9		2	9				1	1	1
	R	5			4		2				3
100	U	20									
	SU	19									1
	CD	8	3		1				1		3
	S	25	1	1	4	2		1		2	
	R	10	2		3					2	
150	U	4		2					1		
	SU	14								2	
	CD	21		2							
	S	7			5			1		2	1
	R	18	1	1	3	2			1		
200	U	26									
	SU	58									
	CD	10								1	
	S	13			8					1	
	R	42	3		5					1	
