# Supplementary material for: Improvement of Predictive Ability by Uniform Coverage of the Target Genetic Space
Source: G3 (Bethesda). 2016 Sep 22;6(11):3733–47. doi: 10.1534/g3.116.035410 (PMC5100872; doi:10.1534/g3.116.035410)
Supplement: Supplemental Material [file supp_g3.116.035410_TableS3.pdf]

Table S3. Dent number of QTLs with a genome-wide significant threshold $p<0.01$ (Li and Ji, 2005). Columns 1-10 represent the linkage groups where the QTLs were located. Multiple QTLs can occur on a chromosome. For the description of the training set construction methods U, SU, CD, S and R see Table 1.

Tasseling

Size	Method	1	2	3	4	5	6	7	8	9	10
50	U		2	4	1				1		1
	SU	1	1	6	2	1	4		3		1
	CD	1	2		6	5		1		2	9
	S	2	1	4	6	7		3	2		1
	R	7	2	3	3	11		2	5	1	1
70	U			8				3	6		
	SU	2		11			1		3		
	CD	2		4	2	12	3	3	10		7
	S	1	1	7	3	4	4		13		
	R	5		6	6	10		2	12	2	2
100	U		2	14		1			29		
	SU			37			2		45		1
	CD	1	1	26	3	8	4		25		2
	S	5		4	4	11	4	4	12	2	8
	R	3	4	10	5	17	7	3	36		1
150	U			92		7	2		98		
	SU	1		74	1	8	2		53	1	
	CD			64	4	4	3		71		4
	S	1		16	9	24	3	3	64	1	1
	R		1	13	7	12	1	15	66	1	2
200	U			54					100		
	SU			60					99		
	CD			52	3	5	1	11	94		
	S		2	19	16	8	1	14	96	1	
	R	1	2	25	10	4	3	11	88		

Silking

Size	Method	1	2	3	4	5	6	7	8	9	10
50	U		1	11	1		1	2		1	1
	SU		1	14	1	1	3		3		
	CD	1	5		3	2			5		
	S	3		4	5	4	3		4	1	1
	R	2	2	2	2	7		1	5	1	
70	U			5				6	2		
	SU	3	2	20				1			
	CD	2	1	2	6	2	5	4	17		2
	S	2	1	6	3	7	7		13	2	
	R	1	3	7	5	6	2	3	12	2	2
100	U			3					8		
	SU			26			1		27		1
	CD		3	15		3	3	2	30		
	S	3	1	3	1	3	2	5	3	1	2
	R	1		5	7	9	10	2	37	1	2
150	U			40					77		
	SU		4	30	1	1	1		38		
	CD	1		17	2				57		2
	S	5	3	6	3	7	4	1	38		1
	R	1	3	4	8	2		10	57		2
200	U			1					99		
	SU			5					97		
	CD			3	1			8	81		
	S	1	10	4	6	1		10	82		
	R	2	6	2	8	3		8	81		1

Yield

Size	Method	1	2	3	4	5	6	7	8	9	10
50	U				1			1	1		
	SU		3		4		1			1	1
	CD					9			2	2	
	S	6	1	1	6		3	4	2	4	8
	R	4	3	3	3	14			2	2	1
70	U	1	2		1			1			
	SU		3		8	4	7	1			
	CD	1	2	1	3	5	1		3		2
	S	2	2	1	6	8	1	8	4	5	27
	R	4	9	3	5	1	1	2	7		1
100	U	1			4	4	10				
	SU				15	1	1				
	CD		8		2	6	1		2		1
	S	2	1	1	3	1			2	11	1
	R	1	13	1		5	2		2	2	2
150	U				1	7					
	SU		1			20					
	CD	1			6	33			1		1
	S		2	1	2	11			2	4	2
	R	1	1		3	15	1		5	6	3
200	U					97					
	SU					95					
	CD				1	69					
	S				5	36			14	1	
	R	2	2		3	32			5	6	
