# Supplementary material for: Improvement of Predictive Ability by Uniform Coverage of the Target Genetic Space
Source: G3 (Bethesda). 2016 Sep 22;6(11):3733–47. doi: 10.1534/g3.116.035410 (PMC5100872; doi:10.1534/g3.116.035410)
Supplement: Supplemental Material [file supp_g3.116.035410_TableS4.pdf]

Table S4. Rice number of QTLs with a genome-wide significant threshold $p<0.01$ (Li and Ji, 2005). Columns 1-10 represent the linkage groups where the QTLs were located. Multiple QTLs can occur on a chromosome. For the description of the training set construction methods U, SU, CD, S and R see Table 1.

Flowering

Size	Method	1	2	3	4	5	6	7	8	9	10	11	12
50	U					2							
	SU	1				5			1				
	CD	2		6	1	5	1						
	S	2	2	3	2		3	2	2	5		1	1
	R	6	3	1	5	1	6	2	5		1	2	3
100	U			2		1	1						6
	SU			1		1	1	1				2	10
	CD	4		5	4	2	2		1	2		2	
	S	7	8	5	3	3	11	3	6	7	2	2	
	R	6	1	4	2	3	7	2	2	7		1	
150	U		3	81	1		1			1			
	SU		4	83				1		2			
	CD	5	1	37			1		3	6			1
	S	7	9	11	2	5	29	2	10	8		3	2
	R	6	5	12	2	4	20	1	13	7	2	4	
200	U			100						4			
	SU	5		100			1			3			
	CD	1	1	100	1	1			3	3			2
	S	10	17	13	1	5	57	1	17	8		5	2
	R	3	2	17	1	1	44	1	10	2		2	1
300	U			100			45		2	1		3	
	SU			100			60		5			1	
	CD		2	100		1	36		18			4	
	S	1	1	69			78		13	1			
	R	1	2	75			78		19	2			

Height

Size	Method	1	2	3	4	5	6	7	8	9	10	11	12
50	U	29		1			2					2	
	SU	26		2								1	
	CD	6	4	2	1	1	1		4				
	S	10	1	1		1	1		1			2	
	R	7			1	2	1			1			1
100	U	96	17				2			6		2	6
	SU	96	22				1			3		2	7
	CD	47	1	1		3	2			6			
	S	50	2	2	1	2	4			3	1		1
	R	49	5	4		4	4		1	3		4	
150	U	106	8				5			6			
	SU	106	10				5	1		11			2
	CD	93	3				2			3			
	S	86	9	1		1	3		2				3
	R	90	9	1		3	2		1	2		3	
200	U	109					14			9			3
	SU	111					16			6			1
	CD	102	11				3	1		5			1
	S	104	11	4		3	4	3	1	2		1	1
	R	96	10	1		2	11	2		3			1
300	U	100					40						
	SU	100					46						
	CD	101	13				22						
	S	106	16			2	10					1	
	R	114	22			1	9	1		2		3	

Seed nr

Size	Method	1	2	3	4	5	6	7	8	9	10	11	12
50	U	2	1	1	2	1							1
	SU		5	4			1						1
	CD	2	2	2	2		2	1	1			1	5
	S	3	3	2	5		3	6		2	2		1
	R	4	4	1	5	3	3	4			1	1	4
100	U	5		13	4	2	1		6	1			
	SU	16		10	3	2	2		9			1	
	CD	2		1	2		3		1				7
	S	1	2	1	3	1	5	6	5	2	1	3	4
	R	2		5	4		4	4				1	4
150	U	10					9			1		1	
	SU	12					4						
	CD	8	1				2	1		2			2
	S	5	3	6	9	1	7	4	2		1	2	9
	R	3		2	5		14	7	3	2		1	13
200	U	3											
	SU	1		4									
	CD	21		1	4		23	1					29
	S	9	2	2	7		7	8		2		6	21
	R	15		4	2		6	8				1	21
300	U			4	7		2						14
	SU			2	14		6						12
	CD	21		1	4		23	1					29
	S	8		4	1		16	9					50
	R	10		3			18	9					47
