# Supplementary material for: Improvement of Predictive Ability by Uniform Coverage of the Target Genetic Space
Source: G3 (Bethesda). 2016 Sep 22;6(11):3733–47. doi: 10.1534/g3.116.035410 (PMC5100872; doi:10.1534/g3.116.035410)
Supplement: Supplemental Material [file supp_g3.116.035410_TableS5.pdf]

Table S5. Flint Tasseling date predictive ability within groups using a training set size of 200 genotypes. For the description of the training set construction methods U, SU, CD, S and R see Table 1. s.e. indicates the mean standard error across methods.

			QTL			
**Subpop.**	**U**	**SU**	**CD**	**S**	**R**	**s.e.**
a	0.141	0.18	0.185	0.145	0.127	0.091
b	0.627	0.832	0.615	0.663	0.684	0.099
c	0.366	0.368	0.535	0.238	0.271	0.058
d	0.272	0.523	0.424	0.711	0.496	0.098
e	0.214	0.106	0.215	0.008	0.063	0.045
			**GBLUP**			
**Subpop.**	**U**	**SU**	**CD**	**S**	**R**	**s.e.**
a	0.316	0.228	0.357	0.269	0.427	0.034
b	0.615	0.849	0.620	0.594	0.649	0.037
c	0.875	0.290	0.899	0.307	0.484	0.026
d	0.802	0.841	0.879	0.526	0.577	0.047
e	0.821	0.555	0.804	0.607	0.548	0.022
			**QGBLUP**			
**Subpop.**	**U**	**SU**	**CD**	**S**	**R**	**s.e.**
a	0.302	0.309	0.372	0.303	0.324	0.071
b	0.608	0.796	0.559	0.632	0.649	0.077
c	0.881	0.359	0.882	0.331	0.432	0.055
d	0.774	0.824	0.769	0.635	0.495	0.098
e	0.847	0.518	0.743	0.423	0.450	0.045
			**RKHS**			
**Subpop.**	**U**	**SU**	**CD**	**S**	**R**	**s.e.**
a	0.460	0.315	0.426	0.239	0.431	0.034
b	0.654	0.944	0.65	0.593	0.655	0.037
c	0.826	0.308	0.856	0.402	0.529	0.026
d	0.771	0.843	0.887	0.539	0.614	0.047
e	0.847	0.562	0.786	0.608	0.540	0.022
