# Supplementary material for: Improvement of Predictive Ability by Uniform Coverage of the Target Genetic Space
Source: G3 (Bethesda). 2016 Sep 22;6(11):3733–47. doi: 10.1534/g3.116.035410 (PMC5100872; doi:10.1534/g3.116.035410)
Supplement: Supplemental Material [file supp_g3.116.035410_TableS6.pdf]

Table S6. Flint Yield predictive ability within groups using a training set size of 200 genotypes. For the description of the training set construction methods U, SU, CD, S and R see Table 1. s.e. indicates the mean standard error across methods.

**Flint, Yield, 200 genotypes**						
			**QTL**			
**Subpop.**	**U**	**SU**	**CD**	**S**	**R**	**s.e.**
a	-0.114	-0.003	-0.146	-0.248	-0.048	0.135
b	0.521	0.015	0.328	0.419	0.473	0.194
c	0.030	0.399	0.130	-0.071	0.061	0.136
d	0.628	0.838	0.638	0.666	0.708	0.102
e	0.555	-0.124	0.331	0.043	0.009	0.038
			**GBLUP**			
**Subpop.**	**U**	**SU**	**CD**	**S**	**R**	**s.e.**
a	0.131	0.279	0.238	0.117	0.372	0.040
b	0.564	0.094	0.516	0.401	0.314	0.053
c	0.403	0.195	0.371	0.236	0.270	0.028
d	0.672	0.619	0.783	0.603	0.600	0.039
e	0.702	0.531	0.662	0.457	0.442	0.016
			**QGBLUP**			
**Subpop.**	**U**	**SU**	**CD**	**S**	**R**	**s.e.**
a	0.022	0.158	0.137	-0.127	0.257	0.090
b	0.670	0.044	0.506	0.457	0.450	0.120
c	0.408	0.266	0.402	0.041	0.217	0.064
d	0.674	0.657	0.771	0.708	0.676	0.089
e	0.687	0.361	0.521	0.183	0.170	0.037
			**RKHS**			
**Subpop.**	**U**	**SU**	**CD**	**S**	**R**	**s.e.**
a	0.142	0.337	0.290	0.245	0.392	0.040
b	0.447	0.037	0.408	0.360	0.271	0.053
c	0.382	0.240	0.342	0.259	0.284	0.028
d	0.708	0.680	0.818	0.679	0.688	0.039
e	0.745	0.451	0.666	0.41	0.389	0.016
