# Supplementary material for: Improvement of Predictive Ability by Uniform Coverage of the Target Genetic Space
Source: G3 (Bethesda). 2016 Sep 22;6(11):3733–47. doi: 10.1534/g3.116.035410 (PMC5100872; doi:10.1534/g3.116.035410)
Supplement: Supplemental Material [file supp_g3.116.035410_TableS7.pdf]

Table S7. Dent Silking date predictive ability within groups using a training set size of 150 genotypes. For the description of the training set construction methods U, SU, CD, S and R see Table 1. s.e. indicates the mean standard error across methods.

**Dent, Silking date, 150 genotypes**						
**QTL**						
**Subpop.**	**U**	**SU**	**CD**	**S**	**R**	**s.e.**
a	0.049	0.060	-0.057	-0.063	-0.031	0.080
b	0.044	-0.072	0.060	0.040	0.090	0.025
c	0.113	0.020	0.213	0.210	0.243	0.028
d	0.179	0.079	0.124	0.212	0.276	0.116
e	0.387	0.212	0.280	0.112	0.161	0.035
f	0.455	0.455	0.374	-	0.337	0.017
**GBLUP**						
**Subpop.**	**U**	**SU**	**CD**	**S**	**R**	**s.e.**
a	0.512	0.532	0.364	0.327	0.376	0.051
b	0.610	0.554	0.597	0.706	0.649	0.019
c	0.526	0.458	0.478	0.410	0.410	0.021
d	0.549	0.541	0.517	0.630	0.521	0.036
e	0.772	0.707	0.681	0.683	0.671	0.025
f	0.820	0.825	0.778	-	0.700	0.014
**QGBLUP**						
**Subpop.**	**U**	**SU**	**CD**	**S**	**R**	**s.e.**
a	0.488	0.570	0.287	0.110	0.108	0.071
b	0.443	0.380	0.431	0.549	0.510	0.025
c	0.532	0.426	0.539	0.462	0.494	0.028
d	0.522	0.503	0.452	0.688	0.547	0.047
e	0.700	0.652	0.609	0.632	0.612	0.032
f	0.712	0.761	0.698	-	0.637	0.017
**RKHS**						
**Subpop.**	**U**	**SU**	**CD**	**S**	**R**	**s.e.**
a	0.492	0.465	0.345	0.131	0.158	0.051
b	0.641	0.537	0.602	0.612	0.573	0.019
c	0.477	0.415	0.412	0.356	0.344	0.021
d	0.618	0.610	0.574	0.600	0.531	0.036
e	0.757	0.699	0.664	0.638	0.650	0.025
f	0.777	0.786	0.717	-	0.628	0.014
