# Supplementary material for: Improvement of Predictive Ability by Uniform Coverage of the Target Genetic Space
Source: G3 (Bethesda). 2016 Sep 22;6(11):3733–47. doi: 10.1534/g3.116.035410 (PMC5100872; doi:10.1534/g3.116.035410)
Supplement: Supplemental Material [file supp_g3.116.035410_TableS8.pdf]

Table S8. Dent Tasseling date predictive ability within groups using a training set size of 150 genotypes. For the description of the training set construction methods U, SU, CD, S and R see Table 1. s.e. indicates the mean standard error across methods.

Dent, Tasseling date, 150 genotypes						
**QTL**						
**Subpop.**	**U**	**SU**	**CD**	**S**	**R**	**s.e.**
a	0.308	0.207	0.236	0.164	0.094	0.019
b	0.201	0.140	0.267	0.290	0.337	0.023
c	0.058	-0.075	0.105	0.162	0.204	0.078
d	0.544	0.435	0.443	0.224	0.221	0.028
e	0.385	0.439	0.225	0.094	0.207	0.067
f	0.513	0.510	0.472	-	0.374	0.014
**GBLUP**						
**Subpop.**	**U**	**SU**	**CD**	**S**	**R**	**s.e.**
a	0.554	0.543	0.511	0.640	0.581	0.018
b	0.445	0.390	0.444	0.410	0.420	0.021
c	0.547	0.574	0.615	0.632	0.553	0.039
d	0.806	0.743	0.718	0.721	0.703	0.025
e	0.595	0.639	0.593	0.486	0.475	0.046
f	0.812	0.817	0.771	-	0.709	0.013
**QGBLUP**						
**Subpop.**	**U**	**SU**	**CD**	**S**	**R**	**s.e.**
a	0.466	0.432	0.414	0.528	0.423	0.019
b	0.530	0.446	0.531	0.518	0.551	0.023
c	0.490	0.508	0.549	0.585	0.536	0.042
d	0.730	0.671	0.658	0.647	0.638	0.028
e	0.654	0.721	0.558	0.328	0.473	0.062
f	0.689	0.729	0.704	-	0.647	0.014
**RKHS**						
**Subpop.**	**U**	**SU**	**CD**	**S**	**R**	**s.e.**
a	0.580	0.558	0.520	0.541	0.501	0.018
b	0.409	0.361	0.368	0.367	0.358	0.021
c	0.618	0.656	0.638	0.598	0.565	0.039
d	0.796	0.735	0.708	0.678	0.680	0.025
e	0.630	0.626	0.614	0.318	0.229	0.046
f	0.759	0.775	0.703	-	0.626	0.013
