# Supplementary material for: Improvement of Predictive Ability by Uniform Coverage of the Target Genetic Space
Source: G3 (Bethesda). 2016 Sep 22;6(11):3733–47. doi: 10.1534/g3.116.035410 (PMC5100872; doi:10.1534/g3.116.035410)
Supplement: Supplemental Material [file supp_g3.116.035410_TableS9.pdf]

Table S9. Dent Yield predictive ability within groups using a training set size of 150 genotypes. For the description of the training set construction methods U, SU, CD, S and R see Table 1. s.e. indicates the mean standard error across methods.

**Dent, Yield, 150 genotypes**						
**QTL**						
**Subpop.**	**U**	**SU**	**CD**	**S**	**R**	**s.e.**
a	0.055	0.275	0.067	0.394	0.045	0.101
b	0.368	0.305	0.266	0.149	0.215	0.040
c	0.299	-0.06	0.361	0.365	0.286	0.133
d	0.424	0.488	0.399	0.378	0.274	0.047
e	0.391	0.455	0.346	0.121	0.331	0.044
f	0.249	0.189	0.312	-	0.202	0.027
**GBLUP**						
**Subpop.**	**U**	**SU**	**CD**	**S**	**R**	**s.e.**
a	-0.193	-0.144	-0.026	0.285	0.037	0.042
b	0.38	0.352	0.434	0.359	0.364	0.017
c	0.385	-0.01	0.544	0.608	0.586	0.053
d	0.578	0.501	0.404	0.477	0.441	0.020
e	0.669	0.688	0.676	0.654	0.65	0.019
f	0.574	0.547	0.539	-	0.493	0.012
**QGBLUP**						
**Subpop.**	**U**	**SU**	**CD**	**S**	**R**	**s.e.**
a	-0.005	0.213	0.072	0.518	0.141	0.096
b	0.478	0.387	0.445	0.321	0.365	0.039
c	0.486	-0.128	0.489	0.516	0.401	0.120
d	0.644	0.604	0.464	0.479	0.462	0.046
e	0.543	0.575	0.609	0.514	0.624	0.044
f	0.604	0.537	0.537	-	0.414	0.027
**RKHS**						
**Subpop.**	**U**	**SU**	**CD**	**S**	**R**	**s.e.**
a	-0.103	-0.08	-0.06	0.268	0.032	0.042
b	0.369	0.340	0.385	0.314	0.311	0.017
c	0.393	0.016	0.625	0.572	0.538	0.053
d	0.438	0.423	0.334	0.418	0.370	0.020
e	0.581	0.588	0.575	0.576	0.564	0.019
f	0.557	0.529	0.522	-	0.455	0.012
