# Supplementary figures and images for: Improvement of Predictive Ability by Uniform Coverage of the Target Genetic Space
Source: G3 (Bethesda). 2016 Sep 22;6(11):3733–47. doi: 10.1534/g3.116.035410 (PMC5100872; doi:10.1534/g3.116.035410)

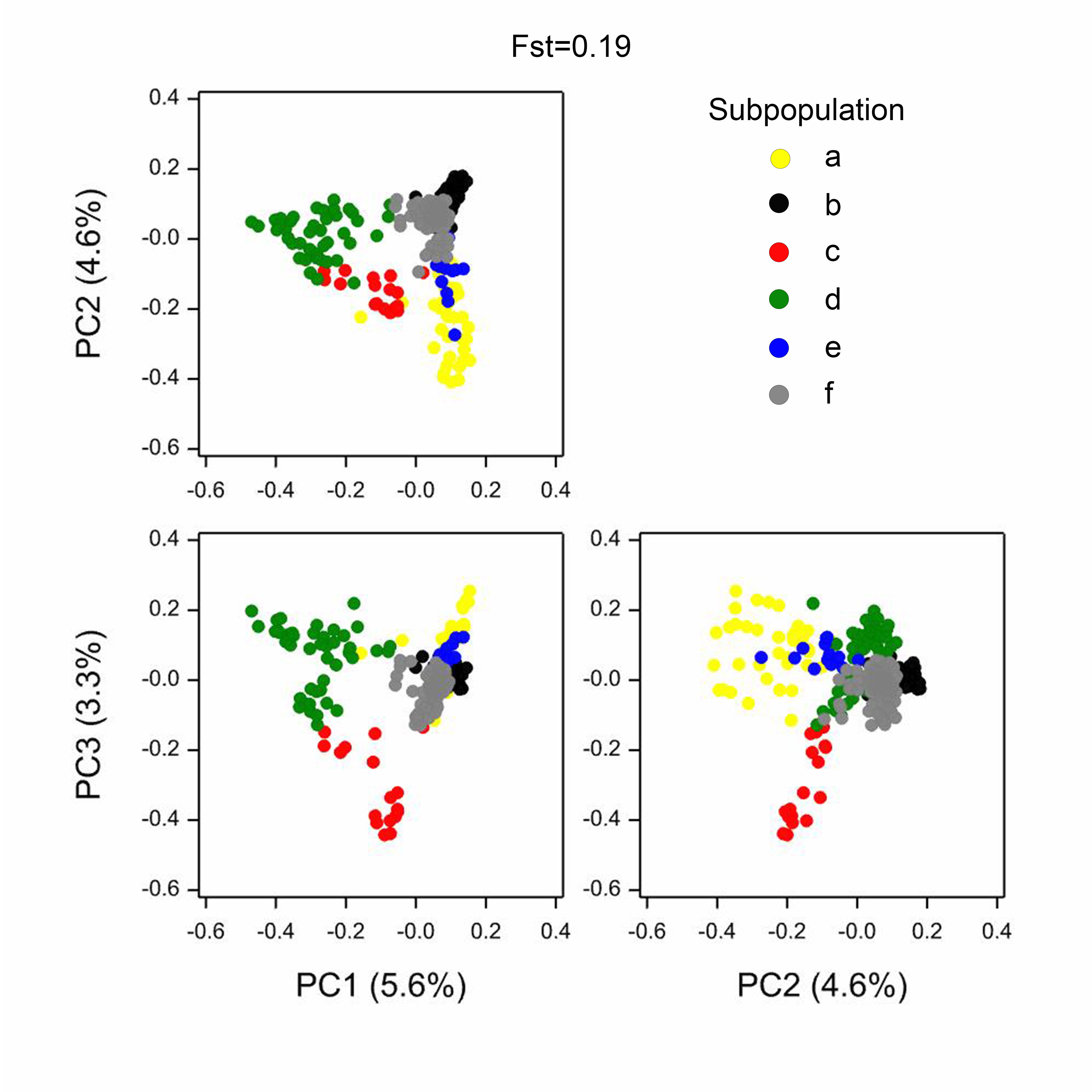

Supplement: Supplemental Material [file supp_g3.116.035410_FigureS1.jpg]

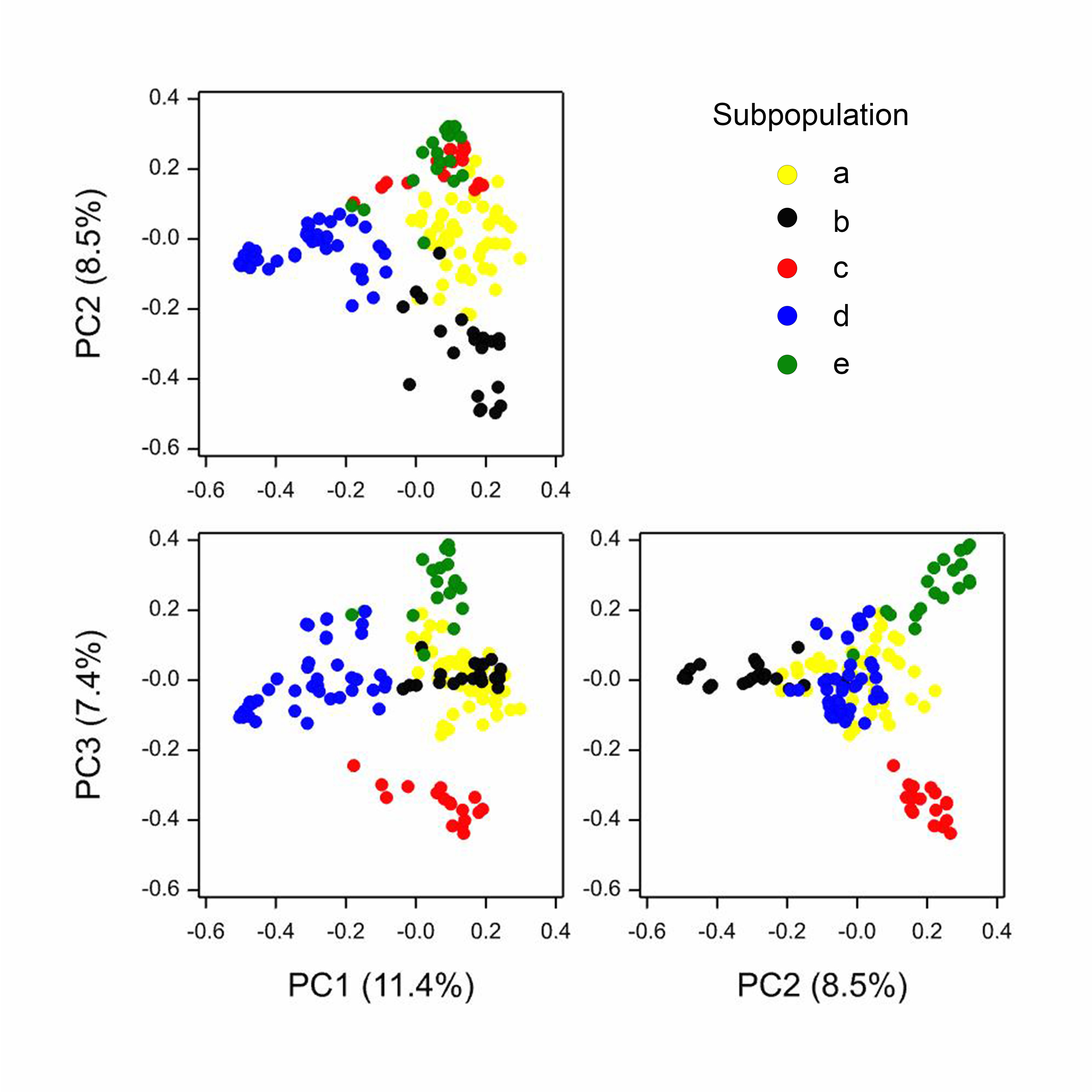

Supplement: Supplemental Material [file supp_g3.116.035410_FigureS2.jpg]
